# Supplementary material for: A gene expression atlas of embryonic neurogenesis in Drosophila reveals complex spatiotemporal regulation of lncRNAs
Source: Development. 2019 Mar 28;146(6):dev175265. doi: 10.1242/dev.175265 (PMC6451322; doi:10.1242/dev.175265)
Supplement: Supplementary information [file develop-146-175265-s1.pdf]

Supplementary Materials and Methods

Fly strains and husbandry

Drosophila melanogaster lines used in this study include

yellow-white	*y^1 w^{1118}*	(source: BSC# 6598)
IC-GFP	*y^1 w^{1118} ; ; P{3xind_1.4-GFP}*	(this study)
IC-dsRed	*y^1 w^{1118} ; P{3xind_1.4-dsRed}*	(this study)
VC-dsRed	*y^1 w^{1118} ; ; P{2xvnd_743-dsRED}*	(Karaiskos et al. 2017)
vnd-lexA/VC:FNLDD	*y^1 w^{1118} ; M{3xvnd-lexA:Cit}$^{attP\_ZH51C}$; P{vnd:FNLDD}*	(Krueger et al., unpublished)
rho-lexA/VC:FNLDD	*y^1 w^{1118} ; M{3xrho-lexA:Cit}$^{attP\_ZH51C}$; P{vnd:FNLDD}*	(Krueger et al., unpublished)

The *IC-GFP* and *IC-dsRed* lines were created by standard P-element transgenesis (Rubin & Spradling 1982) in a *y^1 w^{1118}* background. The *ind* enhancer (*ind_1.4*) (Markstein et al. 2004) was cloned using primers (ngctagcgtcgacGCTTCAAAGCTCCGGGAAACG & nctcgagTCTGGGCCTTCGGTCCGAAAATG) flanked with NheI and SalI restriction sites (F primer) and with XhoI (R primer). PCR product was T/A cloned into pCRII-Duo and concatemerized using the compatibly cohesive sites XhoI and SalI. 3x *ind_1.4* constructs were directionally subcloned into the P-element vectors pH-Stinger or pRed-HStinger (Barolo et al. 2004) using NheI and XhoI.

Fly stocks were maintained at 25°C, ~60% relative humidity on standard fly food with 12 hr light/dark cycles according to standard procedures. 2-hr embryo collections were done on apple juice agar plates with yeast paste after 3 x 1hr pre-lays in the morning, embryos were then aged for an appropriate amount of time at 25°C and ~60% relative humidity before dechorionation.

DIV-SortSeq

This protocol incorporates portions from MARIS (Hrvatin et al. 2014). All steps after dechorionation were performed on ice, using ice-cold DEPC-treated solutions. Primary and secondary antibodies used in this study are listed in Table S1. RNase-free BSA was obtained from Gemini Bioproducts and is critical for isolation of high-quality RNA.

Embryos were collected, dechorionated, and dissociated into single-cell suspension in 15ml PBS, pH 7.5 using 8-12 strokes with the loose pestle of a glass dounce homogenizer. Large debris was removed via filtration with 2 x 90°-rotated sheets of Miracloth into a 15ml conical vial, followed by centrifugation at 40x*g*, 4°C, 3 min. The supernatant was transferred to a 15ml conical vial, cells were pelleted at 1000x*g*, 4°C, 3 min, washed with 1ml PBS, and fixed with 4% formaldehyde at 4°C, 15min. Cross-linking was stopped with Quench Buffer (2.5M Glycine in PBS). Fixed cells were washed twice with 1ml ice-cold PBS, and stored at 4°C overnight in 1ml RNA*later*™ (Thermo, AM7020). Cells were rehydrated via dilution in 9ml ice-cold PBS, centrifugation at 1000 x*g*, 10min, and washed twice in 1ml ice-cold PBS. Fixed cell suspensions were immunostained under RNase-free conditions with primary antibodies with agitation at 4 °C, 1.5-2h in 250µl Stain Buffer (1% BSA (w/v), 0.1% saponin, 1:200 RNase inhibitor in RNase-free

Development • Supplementary information

PBS). After washing 3x 5 min in 1ml Wash Buffer (0.2% BSA, 0.1% saponin in PBS), cells were incubated with conjugated secondary antibodies with agitation at 4°C, 45 min-1h in 250µl Stain Buffer. After washing 3x 5 min in 1ml Wash Buffer, cells were resuspended in 1ml Sort Buffer (0.5% BSA, 2mM EDTA, 1:500 RNase inhibitor in PBS) and filtered with a 70µm cell strainer. Filtered cells were subjected to FACS purification on the FACSAria II (BD Biosciences) and sorted cells collected in Collection Buffer (2% BSA, 1:100 RNase inhibitor in PBS).

FACS-purified cells were pelleted and resuspended in 200µl Digestion Buffer (5M NaCl, 1M Tris-HCl pH 8.0, 200mM EDTA, 10% SDS, 3.2U Proteinase K, 1:100 RNase inhibitor) and incubated at 50°C, 15min (Proteinase K digestion) followed by 80°C, 15min (reversal of formaldehyde cross-links). Samples were transferred to ice and resuspended in 600µl TRIzol™ LS Reagent (Thermo, 10296028). RNA was isolated using the DirectZol™ RNA MicroPrep Kit (Zymo Research, R2060) according to the manufacturer's instructions. RNA concentration was measured using the Qubit™ RNA HS Assay kit (Thermo, Q32852) and RNA quality determined using the Agilent RNA 6000 Pico Kit (Agilent, 5067-1513). All RNA-seq libraries were constructed using the NuGEN Ovation *Drosophila* RNA-Seq System with 10 ng – 100 ng total RNA input. Library concentration was quantified using the Qubit™ dsDNA HS Assay (Thermo, Q32854) and quality was determined on a BioAnalyzer™ using Agilent High Sensitivity DNA Kits (Agilent, 5067-4626). All libraries were sequenced on the Illumina HiSeq4000 at a mean depth of 62.5 million 75bp paired-end reads per sample. RNA-seq datasets generated for this study are detailed in Tables S5 and S6. A detailed, step-wise protocol is available upon request.

Nuclear-cytoplasmic fractionation

The cell fractionation procedure incorporates portions from MARIS (Hrvatin et al. 2014). All steps were performed on ice, using ice-cold DEPC-treated solutions, and all centrifugation steps were performed at 4˚C. Embryos were processed to single-cell suspension as described above for *DIV*-SortSeq, then pelleted and resuspended in Cyto Extract Buffer (20mM Tris pH 7.6, 0.1mM EDTA, 2mM $MgCl_2$). After hypotonic swelling, cells were gently lysed by addition of 0.6% CHAPS for isolation of the cytoplasmic fraction. Nuclei were pelleted at 500x*g* for 5min, and the supernatant retained, and an appropriate volume of TRIzol™ LS Reagent was added (cytoplasmic fraction).

Nuclei were washed with Nuclei Wash Buffer (20mM Tris pH 7.6, 0.1mM EDTA, 2mM $MgCl_2$, 0.6% CHAPS), resuspended in Nuclei Resuspension Buffer (10mM Tris, pH 7.6, 150mM NaCl, 0.15% NP-40) and pelleted at 12,000x*g*, 10min in Sucrose Buffer (10mM Tris, pH 7.5, 150mM NaCl, 24% sucrose). After washing (1mM EDTA in PBS), nuclei pellet was resuspended in TRIzol™ Reagent. RNA was isolated and concentration and quality determined as described for above for *DIV*-SortSeq.

Quantitative RT-PCR (qPCR)

50ng of total RNA was reverse-transcribed with the QuantiTect Reverse Transcription kit (Qiagen, 205310) in a total volume of 20µl, according to the manufacturer's instructions. For each gene, 0.2µl

Development • Supplementary information

cDNA was used for input into qPCR using SensiFAST™ SYBR® No-ROX Kit (Bioline, 98020) and 5µM forward and reverse primers in a total volume of 20µl. qPCR primer sequences are listed in Table S2. qPCR thermal cycling and fluorescent data acquisition was performed using the BioRad CFX96 Touch™ Real-Time PCR Detection System. Expression fold changes were calculated via the $\Delta\Delta C_T$ method (Vandesompele et al. 2002; Schmittgen & Livak 2008), normalized to the mean C_T of two reference genes: *α-tubulin* and *actin 42A*.

RNA probe design and synthesis for RNA in situ hybridization.

Where available, cDNA clones were obtained from the *Drosophila* Gene Collection or *Drosophila* Genomics Resource Center (Stapleton et al. 2002), detailed in Table S3. Constructs were linearized via restriction digestion, and subjected to *in vitro* transcription using appropriate RNA Polymerases (Roche), using ribonucleotide mixtures containing dUTP-DIG, -FITC, or -Biotin (Roche).

Where cDNA clones were not available, PCR primers were designed to amplify a region within the transcribed locus from genomic DNA. Primer sequences are detailed in Table S4. A T7 promoter sequence appended to the reverse primer allowed *in vitro* transcription directly from the PCR product. After template digest by DNaseI, RNA probes were sheared at 65°C for 3-20 min in carbonation buffer (120mM Na_2CO_3, 80mM $NaHCO_3$, pH 10.2), length of carbonation depended on probe length. RNA probes were precipitated at -20°C overnight and resuspended in Hyb-A Buffer (50% formamide, 5X SSC, 0.1% Tween-20).

Development • Supplementary information

Supplementary Tables

Table S1. VST counts by gene across datasets

Click here to Download Table S1

Table S2. Neurogenic coding genes

Click here to Download Table S2

Table S3. Enriched lncRNAs

Click here to Download Table S3

Table S4. Length-scaled TPMs

Click here to Download Table S4

Table S5. PhyloCSF analysis

Click here to Download Table S5

Column interpretation for Table S5: PhyloCSF

Intervals	Intervals of the input transcript
Strand	Strand
TranscriptName	Name from the input bed file
TrUCSCview	Link to show the entire transcript in UCSC browser
ORFintervals	Intervals of the ORF (not including the stop codon)
ORFstart	0-based transcript coordinate of first base of ORF
ORFend	0-based transcript coordinate of last base of ORF
NumCodons	Number of codons in the ORF
PhyloCSF	Raw PhyloCSF score of the ORF
RelBL	Fraction of branch length of the phylogenetic tree spanned by species present in the alignment of this ORF
ScorePerCodon	PhyloCSF divided by NumCodons
PhyloCSFPsi	Length adjusted score, a log likelihood, in decibans
Pval	Probability a region of this length, none-of-which has ever been coding, has this score or higher
CorrectedPvalTr	p-val with Holm-Bonferroni correction for number of ORFs in this transcript
CorrectedPvalAll	p-val with Holm-Bonferroni correction for total number of ORFs
FDR	Benjamini & Hochberg false discovery rate.
LocalFDR	Local FDR (Efron et al. 2001).
AntiScorePerCodon	Score on opposite strand in frame that shares 3rd codon position
ScoreDiff	ScorePerCodon - AntiScorePerCodon
GC	GC content of ORF
CpGratio	Number of CpGs in ORF divided by the expected number based on C and G content
CodAlignView	Link to view ORF alignment in CodAlignView, the Codon Alignment Viewer, with 10-codon context on each side
OrfUCSCview	Link to show the ORF in UCSC browser

Development • Supplementary information

Table S6: Primary and secondary antibodies used in this study

Target	*Supplier*	*Catalog #*	*Application*	*Antibody Type*	*Conjugate*	*Dilution*
RFP	Thermo	710530	*Primary*	Rabbit polyclonal	N/A	1:500
GFP	Thermo	G10362	*Primary*	Rabbit polyclonal	N/A	1:500
Pros	DSHB	MR1A	*Primary*	Mouse monoclonal	N/A	1:20
Elav	DSHB	9F8A9	*Primary*	Mouse monoclonal	N/A	1:500
Repo	DSHB	8D12	*Primary*	Mouse monoclonal	N/A	1:20
Rabbit IgG	Thermo	A21428	*Secondary*	Goat polyclonal	*Alexa Fluor 555*	1:500
Mouse IgG	Thermo	A32727	*Secondary*	Goat polyclonal	*Alexa Fluor 555*	1:500

Table S7: qPCR primer sequences

Target	*Forward primer*	*Reverse primer*	*Size (bp)*
α-Tubulin	TGTCGCGTGTGAAACACTTC	AGCAGGCGTTTCCAATCTG	585
Actin 42A	GCGTCGGTCAATTCAATCTT	AAGCTGCAACCTCTTCGTCA	292
Prospero	CGGCATGGCTCCTACTTCTT	TAGCGCACCCAGAAGAACAT	78
Worniu	ATGGATAAACTCAAGTACAGCCG	AAGTCCACTGGTCCTTCATCA	107
Elav	ACGCTCCTGCCACAGAAAAA	CGTCGCCGTATTTCGCTC	211
Lim3	GATGGAGGATCGTAAGCTGATCT	GTAGGCCGTTTTCAGGGTCTC	154
Repo	CTCCGCCAAGTAGTTCCTCC	AGGCAGTAAAGGTGGTTCTCG	216
Gcm	ACAAGGCCAGAAGGAAGCAG	CAAGCCTGGATTTCCAAGCGA	76

Development • Supplementary information

Table S8: List of commercially-available cDNA clones for RNA probe synthesis

RNA target	*DGRC Reference #*
ind	RT01026
vnd	PCSP6029
pros	LD37627
elav	LD33076-IR
repo	GH05443-dg
CR30009	RE30084
CR32730	RE54940
CR32111	RE52337

Table S9: PCR primer sequences for RNA probe synthesis

Target	*Forward primer*	*T7 promoter + Reverse primer*	*Size (bp)*
CR46003	TGTGTCGCACAGGATGTGT	TAATACGACTCACTATAGGTGCTGGCGGGGAAATTATGT	908
cherub	CGAGGAACCTTCGGTGCATA	TAATACGACTCACTATAGGGCTTGGGTGATTTCGAGGGA	1511
CR44024	GTGTCGTGTCGGGTAAGTGT	TAATACGACTCACTATAGGAAGTGGCCTGTCTCAGAACG	1268
CR32111	GTATGCGCTCGAACTCGGTAA	TAATACGACTCACTATAGGGCCGGCATGAGCAAACACAAA	1232

Development • Supplementary information

Table S10: Summary of RNA-seq datasets – DIV-SortSeq

Sample name	***Fly line***	***Target protein***	***Cell Type***	***Enriched/depleted***	***Time point***	***Replicate***	***# reads (M)***
4-6h_Ind-neg_1	*IC*-dsRed	dsRed	Intermediate column	Depleted	4-6h	1	89.9
4-6h_Ind-neg_2	*IC*-dsRed	dsRed	Intermediate column	Depleted	4-6h	2	140.1
4-6h_Ind-pos_1	*IC*-dsRed	dsRed	Intermediate column	Enriched	4-6h	1	76.2
4-6h_Ind-pos_2	*IC*-dsRed	dsRed	Intermediate column	Enriched	4-6h	2	81.6
4-6h_Vnd-neg_1	*VC*-dsRed	dsRed	Ventral column	Depleted	4-6h	1	62.6
4-6h_Vnd-neg_2	*VC*-dsRed	dsRed	Ventral column	Depleted	4-6h	2	64.3
4-6h_Vnd-pos_1	*VC*-dsRed	dsRed	Ventral column	Enriched	4-6h	1	61.4
4-6h_Vnd-pos_2	*VC*-dsRed	dsRed	Ventral column	Enriched	4-6h	2	65.6
4-6h_Pros-neg_1	*vnd*-lexA/VC:FNLDD	Prospero	Neuroblasts	Depleted	4-6h	1	59.8
4-6h_Pros-neg_2	*vnd*-lexA/VC:FNLDD	Prospero	Neuroblasts	Depleted	4-6h	2	79.1
4-6h_Pros-pos_1	*vnd*-lexA/VC:FNLDD	Prospero	Neuroblasts	Enriched	4-6h	1	61.9
4-6h_Pros-pos_2	*vnd*-lexA/VC:FNLDD	Prospero	Neuroblasts	Enriched	4-6h	2	59.1
6-8h_Ind-neg_1	*IC*-GFP	GFP	Intermediate column	Depleted	6-8h	1	61
6-8h_Ind-neg_2	*IC*-GFP	GFP	Intermediate column	Depleted	6-8h	2	59.9
6-8h_Ind-pos_1	*IC*-GFP	GFP	Intermediate column	Enriched	6-8h	1	57
6-8h_Ind-pos_2	*IC*-GFP	GFP	Intermediate column	Enriched	6-8h	2	70.9
6-8h_Vnd-neg_1	*VC*-dsRed	dsRed	Ventral column	Depleted	6-8h	1	73.2
6-8h_Vnd-neg_2	*VC*-dsRed	dsRed	Ventral column	Depleted	6-8h	2	61
6-8h_Vnd-pos_1	*VC*-dsRed	dsRed	Ventral column	Enriched	6-8h	1	61.6
6-8h_Vnd-pos_2	*VC*-dsRed	dsRed	Ventral column	Enriched	6-8h	2	73.9
6-8h_Pros-neg_1	*vnd*-lexA/VC:FNLDD	Prospero	Neuroblasts	Depleted	6-8h	1	64.5
6-8h_Pros-neg_2	*vnd*-lexA/VC:FNLDD	Prospero	Neuroblasts	Depleted	6-8h	2	76.6
6-8h_Pros-pos_1	*vnd*-lexA/VC:FNLDD	Prospero	Neuroblasts	Enriched	6-8h	1	54.3
6-8h_Pros-pos_2	*vnd*-lexA/VC:FNLDD	Prospero	Neuroblasts	Enriched	6-8h	2	58.8
6-8h_Elav-neg_1	*IC*-GFP	Elav	Neurons	Depleted	6-8h	1	62.2
6-8h_Elav-neg_2	*yw*	Elav	Neurons	Depleted	6-8h	2	57.2
6-8h_Elav-pos_1	*IC*-GFP	Elav	Neurons	Enriched	6-8h	1	53.8
6-8h_Elav-pos_2	*yw*	Elav	Neurons	Enriched	6-8h	2	70.4
6-8h_Repo-neg_1	*IC*-GFP	Repo	Glia	Depleted	6-8h	1	59.6
6-8h_Repo-neg_2	*IC*-GFP	Repo	Glia	Depleted	6-8h	2	71.4
6-8h_Repo_pos_1	*IC*-GFP	Repo	Glia	Enriched	6-8h	1	66.6
6-8h_Repo_pos_2	*IC*-GFP	Repo	Glia	Enriched	6-8h	2	56.8
8-10h_Elav-neg_1	*y w*	Elav	Neurons	Depleted	8-10h	1	74.5
8-10h_Elav-neg_2	*y w*	Elav	Neurons	Depleted	8-10h	2	44.1
8-10h_Elav-pos_1	*y w*	Elav	Neurons	Enriched	8-10h	1	69.9
8-10h_Elav-pos_2	*y w*	Elav	Neurons	Enriched	8-10h	2	62.9
8-10h_Repo-neg_1	*vnd*-lexA/VC:FNLDD	Repo	Glia	Depleted	8-10h	1	61.5
8-10h_Repo-neg_2	*vnd*-lexA/VC:FNLDD	Repo	Glia	Depleted	8-10h	2	60.9
8-10h_Repo-pos_1	*vnd*-lexA/VC:FNLDD	Repo	Glia	Enriched	8-10h	1	61.4

Development • Supplementary information

8-10h_Repo-pos_2	*vnd*-lexA/VC:FNLDD	Repo	Glia	Enriched	8-10h	2	65.6
18-22h_Elav-neg_1	*vnd*-lexA/VC:FNLDD	Elav	Neurons	Depleted	18-22h	1	68.6
18-22h_Elav-neg_2	*vnd*-lexA/VC:FNLDD	Elav	Neurons	Depleted	18-22h	2	61.9
18-22h_Elav-pos_1	*vnd*-lexA/VC:FNLDD	Elav	Neurons	Enriched	18-22h	1	66
18-22h_Elav-pos_2	*vnd*-lexA/VC:FNLDD	Elav	Neurons	Enriched	18-22h	2	63.9
18-22h_Repo-neg_1	*vnd*-lexA/VC:FNLDD	Repo	Glia	Depleted	18-22h	1	58.3
18-22h_Repo-neg_2	*vnd*-lexA/VC:FNLDD	Repo	Glia	Depleted	18-22h	2	63.4
18-22h_Repo-pos_1	*vnd*-lexA/VC:FNLDD	Repo	Glia	Enriched	18-22h	1	70.5
18-22h_Repo-pos_2	*vnd*-lexA/VC:FNLDD	Repo	Glia	Enriched	18-22h	2	75.9

Table S11: Summary of RNA-seq datasets – Fractionation-Seq

Sample name	***Fly line***	***Fraction***	***Time point***	***Replicate***	***Number of reads (M)***
Cyto_18-22h_1	*rho*-lexA/VC:FNLDD	Cytoplasmic	18-22h	1	163.2
Cyto_18-22h_2	*rho*-lexA/VC:FNLDD	Cytoplasmic	18-22h	2	179.1
Cyto_6-8h_1	*rho*-lexA/VC:FNLDD	Cytoplasmic	6-8h	1	189.3
Cyto_6-8h_2	*rho*-lexA/VC:FNLDD	Cytoplasmic	6-8h	2	179.3
Nuc_18-22h_1	*rho*-lexA/VC:FNLDD	Nuclear	18-22h	1	203
Nuc_18-22h_2	*rho*-lexA/VC:FNLDD	Nuclear	18-22h	2	176.1
Nuc_6-8h_1	*rho*-lexA/VC:FNLDD	Nuclear	6-8h	1	193.9
Nuc_6-8h_2	*rho*-lexA/VC:FNLDD	Nuclear	6-8h	2	193.7
Whole_18-22h_1	*rho*-lexA/VC:FNLDD	Whole embryo	18-22h	1	183.2
Whole_18-22h_2	*rho*-lexA/VC:FNLDD	Whole embryo	18-22h	2	180.7
Whole_6-8h_1	*rho*-lexA/VC:FNLDD	Whole embryo	6-8h	1	197.4
Whole_6-8h_2	*rho*-lexA/VC:FNLDD	Whole embryo	6-8h	2	196.1

Table S12: All protein-coding 'computed genes' correlated (r > 0.9) with neurogenic marker genes in DIV-SortSeq expression data

Flybase ID	*Annotation ID*
**FBgn0036725**	CG18265
**FBgn0034009**	CG8155
**FBgn0036008**	CG3408
**FBgn0030017**	CG2278
**FBgn0264449**	CG43867
**FBgn0051235**	CG31235
**FBgn0037166**	CG11426
**FBgn0032752**	CG10702
**FBgn0038114**	CG11670
**FBgn0037206**	CG12768
**FBgn0032485**	CG9426
**FBgn0038321**	CG6218
**FBgn0038720**	CG6231
**FBgn0033283**	CG11635
**FBgn0043806**	CG32032
**FBgn0033287**	CG8701
**FBgn0031540**	CG3238
**FBgn0031961**	CG7102
**FBgn0040984**	CG4440
**FBgn0026876**	CG11403
**FBgn0035689**	CG7376
**FBgn0025388**	CG12179
**FBgn0036503**	CG13454
**FBgn0040809**	CG13465
**FBgn0032489**	CG15480
**FBgn0041702**	CG15107
**FBgn0034403**	CG18190
**FBgn0031070**	CG12702
**FBgn0250754**	CG42232
**FBgn0261538**	CG42662
**FBgn0034514**	CG13427
**FBgn0036670**	CG13029
**FBgn0031252**	CG13690
**FBgn0040346**	CG3704
**FBgn0039013**	CG4813
**FBgn0037633**	CG9839
**FBgn0028506**	CG4455
**FBgn0037844**	CG4570
**FBgn0037924**	CG14712
**FBgn0035464**	CG12006
**FBgn0027602**	CG8611
**FBgn0037051**	CG10565
**FBgn0052756**	CG32756
**FBgn0036710**	CG6479
**FBgn0039735**	CG7911
**FBgn0023515**	CG14814
**FBgn0033160**	CG11107
**FBgn0033169**	CG11123
**FBgn0029672**	CG2875
**FBgn0025633**	CG13366
**FBgn0050183**	CG30183
**FBgn0030768**	CG9723
**FBgn0025626**	CG4281
**FBgn0035213**	CG2199
**FBgn0031403**	CG15387
**FBgn0043456**	CG4747
**FBgn0052428**	CG32428
**FBgn0031062**	CG14230
**FBgn0035315**	CG8960
**FBgn0032050**	CG13096
**FBgn0038552**	CG18012
**FBgn0031764**	CG9107
**FBgn0035878**	CG7182
**FBgn0034073**	CG8414
**FBgn0033766**	CG8771
**FBgn0037958**	CG6962
**FBgn0034447**	CG7744
**FBgn0035842**	CG7504
**FBgn0038272**	CG7265
**FBgn0039544**	CG12877
**FBgn0033615**	CG7741
**FBgn0030122**	CG16892
**FBgn0051365**	CG31365
**FBgn0052318**	CG32318
**FBgn0039566**	CG4849
**FBgn0037746**	CG8478
**FBgn0030813**	CG4949
**FBgn0029825**	CG12728
**FBgn0038768**	CG4936
**FBgn0037149**	CG14561
**FBgn0037372**	CG2091
**FBgn0030915**	CG6179
**FBgn0027514**	CG1024
**FBgn0031947**	CG7154
**FBgn0030660**	CG8097
**FBgn0036565**	CG5235
**FBgn0024364**	CG11417
**FBgn0036994**	CG5199
**FBgn0033527**	CG11777
**FBgn0039743**	CG7946
**FBgn0262719**	CG43163
**FBgn0036886**	CG9300
**FBgn0034264**	CG10933
**FBgn0025627**	CG4194
**FBgn0032682**	CG10176
**FBgn0031001**	CG7884
**FBgn0037213**	CG12581
**FBgn0038660**	CG14291
**FBgn0034933**	CG3735
**FBgn0030855**	CG5800
**FBgn0035414**	CG14965
**FBgn0036214**	CG7264
**FBgn0030317**	CG1561
**FBgn0036660**	CG13025
**FBgn0031597**	CG17612
**FBgn0036483**	CG12316
**FBgn0032512**	CG9305
**FBgn0037644**	CG11964
**FBgn0040385**	CG12496
**FBgn0033802**	CG17724
**FBgn0039733**	CG11504
**FBgn0037504**	CG1142
**FBgn0036522**	CG7372
**FBgn0035402**	CG12082
**FBgn0031769**	CG9135
**FBgn0038551**	CG7357
**FBgn0032348**	CG4751
**FBgn0035987**	CG3689
**FBgn0037918**	CG6791
**FBgn0035872**	CG7185
**FBgn0031529**	CG9662
**FBgn0031492**	CG3542
**FBgn0086855**	CG17078
**FBgn0033990**	CG10265
**FBgn0032454**	CG5787
**FBgn0037622**	CG8202
**FBgn0032751**	CG17343
**FBgn0266917**	CG16941
**FBgn0034114**	CG4282
**FBgn0034750**	CG3732
**FBgn0030738**	CG9915
**FBgn0035235**	CG7879
**FBgn0028474**	CG4119
**FBgn0085451**	CG34422
**FBgn0030293**	CG1737
**FBgn0050020**	CG30020
**FBgn0029941**	CG1677
**FBgn0033021**	CG10417
**FBgn0027503**	CG11970
**FBgn0032388**	CG6686
**FBgn0038546**	CG7379
**FBgn0035481**	CG12605
**FBgn0260451**	CG14042
**FBgn0035677**	CG13293
**FBgn0036202**	CG6024
**FBgn0035643**	CG13287
**FBgn0034184**	CG9646
**FBgn0052105**	CG32105
**FBgn0031257**	CG4133
**FBgn0037050**	CG10566
**FBgn0025712**	CG13920
**FBgn0027550**	CG6495
**FBgn0031762**	CG9098
**FBgn0035246**	CG13928
**FBgn0039808**	CG12071
**FBgn0030508**	CG15760
**FBgn0033960**	CG10151
**FBgn0030596**	CG12398
**FBgn0052085**	CG32085
**FBgn0030012**	CG18262
**FBgn0030223**	CG2111
**FBgn0051030**	CG31030
**FBgn0035903**	CG6765
**FBgn0030595**	CG14406
**FBgn0033983**	CG10253
**FBgn0031955**	CG14535
**FBgn0033497**	CG12912
**FBgn0034154**	CG5267
**FBgn0033872**	CG6329
**FBgn0030588**	CG9521
**FBgn0085382**	CG34353
**FBgn0085218**	CG34189
**FBgn0030594**	CG9509
**FBgn0034459**	CG16716
**FBgn0259823**	CG42404
**FBgn0032800**	CG10137
**FBgn0038926**	CG13409
**FBgn0039064**	CG4467
**FBgn0259163**	CG42268
**FBgn0259994**	CG42492
**FBgn0039030**	CG6660
**FBgn0036579**	CG5027
**FBgn0029708**	CG3556
**FBgn0030586**	CG12539
**FBgn0038596**	CG14312
**FBgn0263072**	CG43347
**FBgn0037525**	CG17816
**FBgn0030742**	CG9919
**FBgn0265084**	CG44195
**FBgn0030592**	CG9514
**FBgn0262476**	CG43066
**FBgn0035033**	CG3548
**FBgn0040351**	CG11638
**FBgn0028647**	CG11902
**FBgn0031313**	CG5080
**FBgn0039915**	CG1732
**FBgn0029896**	CG3168
**FBgn0039024**	CG4721
**FBgn0032897**	CG9336
**FBgn0034128**	CG4409
**FBgn0034417**	CG15117
**FBgn0031816**	CG16947
**FBgn0030261**	CG15203
**FBgn0033446**	CG1648
**FBgn0052354**	CG32354
**FBgn0031627**	CG15630
**FBgn0036927**	CG7433
**FBgn0034618**	CG9485
**FBgn0083972**	CG34136
**FBgn0031589**	CG3714
**FBgn0036760**	CG5567
**FBgn0032899**	CG9338

Supplementary Figures

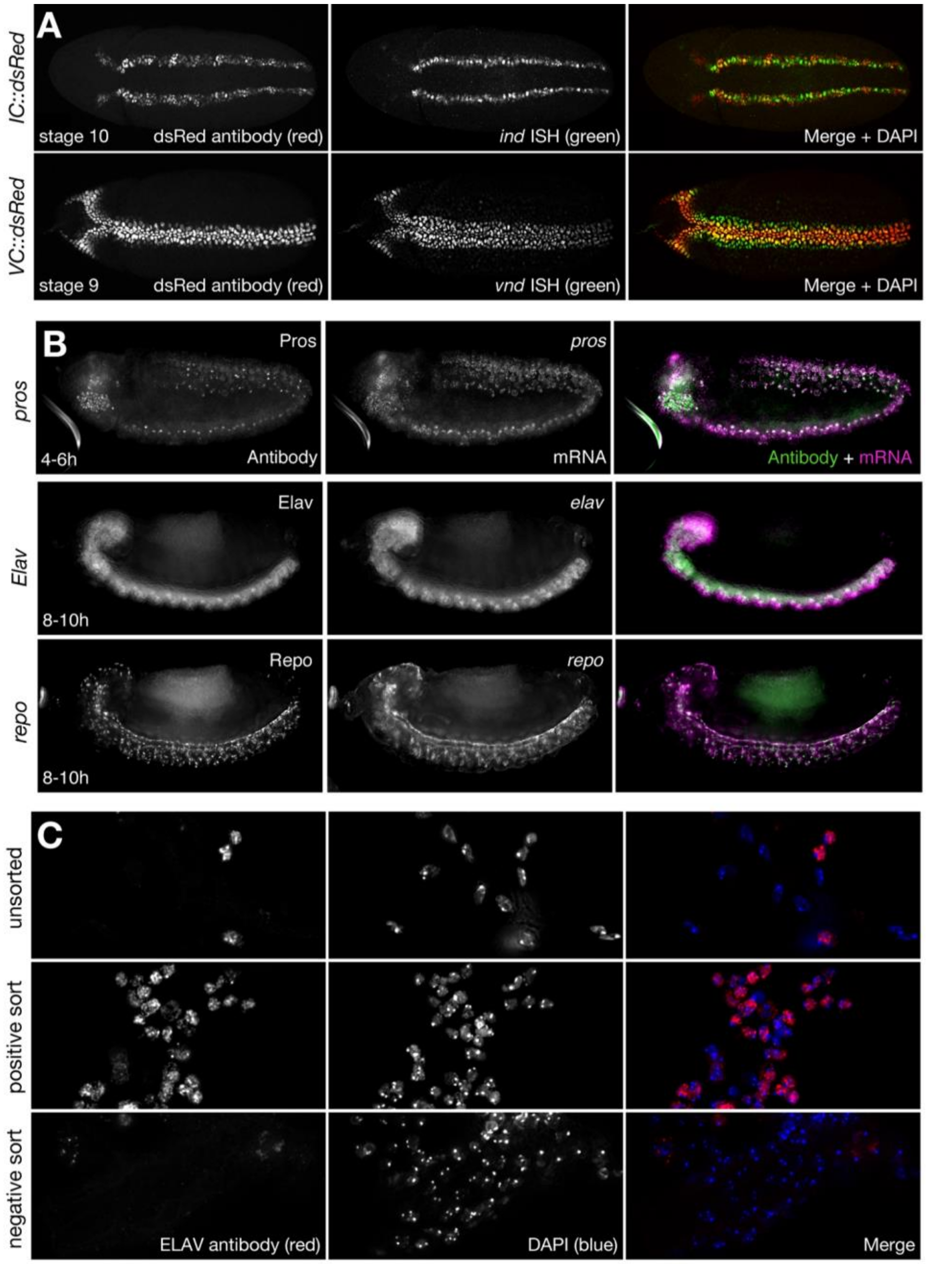

Fig. S1: Neurogenic cell populations can be faithfully marked and purified
(A) Visualization of fluorescent reporter expression in *VC-dsRed* (top panel) and *IC-dsRed* (bottom panel) transgenic embryos. Tissue reporter shown on left and in red by antibody stain, expression of the tissue marker is shown in the middle and in green by *in situ* hybridization. Shown are whole mount embryos, ventral views anterior left. (B) Multiplex whole mount immunohistochemistry (green) and RNA-FISH (magenta) show faithfulness of the antibody sorting markers for neuroblasts (Pros; top panel) at 4-6h, and neurons (Elav; middle panel) and glia (Repo; bottom panel) at 8-10h. (C) Immunohistochemical staining of endogenous Elav protein in cells of dissociated embryos pre- and post-FACS.

Development • Supplementary information

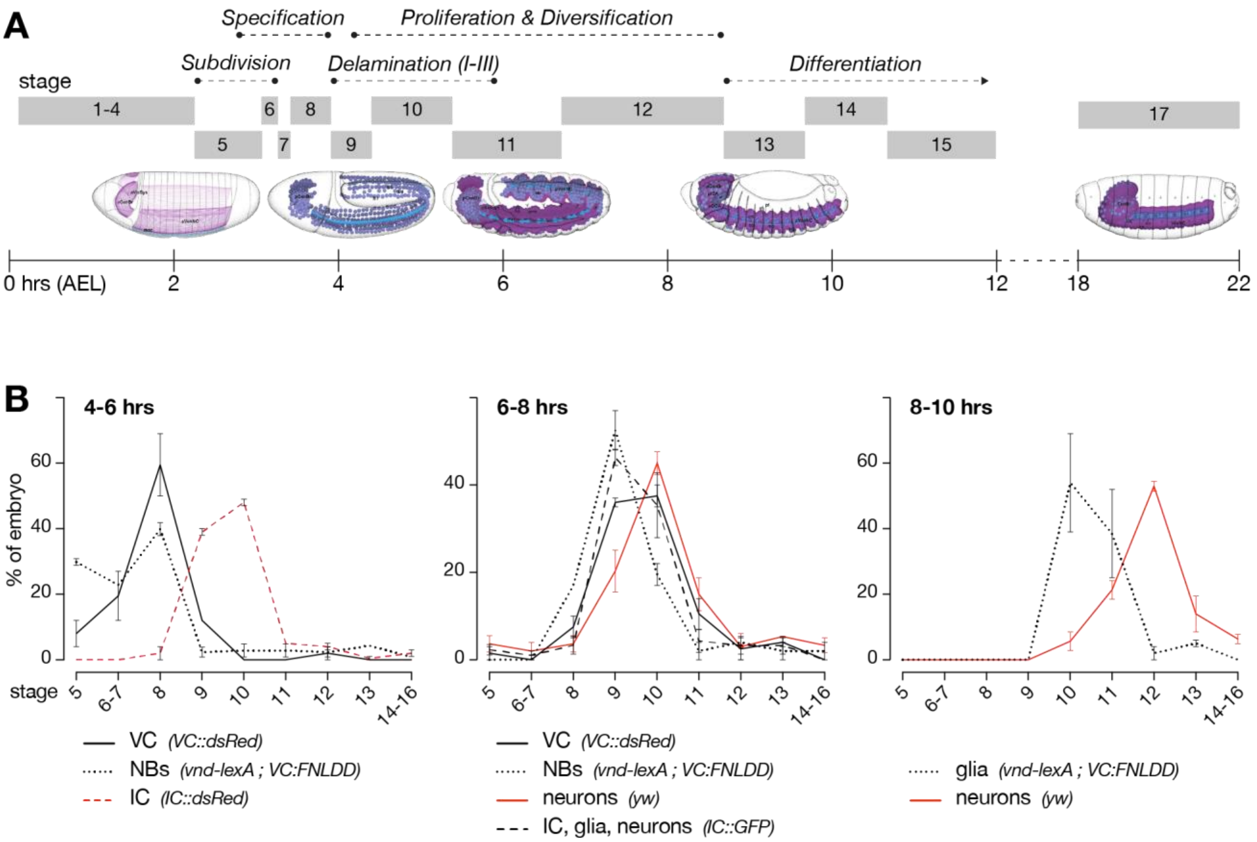

Fig. S2: Timed embryo collections encompass major neurogenic events
(A) Overview of *Drosophila* embryonic neurogenesis, timing and stages. Embryo images show stages of neurogenesis schematically and were obtained from the *Atlas of Drosophila Development* by Volker Hartstein (CSHL Press, 1992, used with permission). (B) Staging of representative samples corresponding to timed embryo collections (n = 2-3 collections per line). Staging according to (Campos-Ortega & Hartenstein 1985). Cell type markers targeted for *DIV*-MARIS indicated for each fly line.

Development • Supplementary information

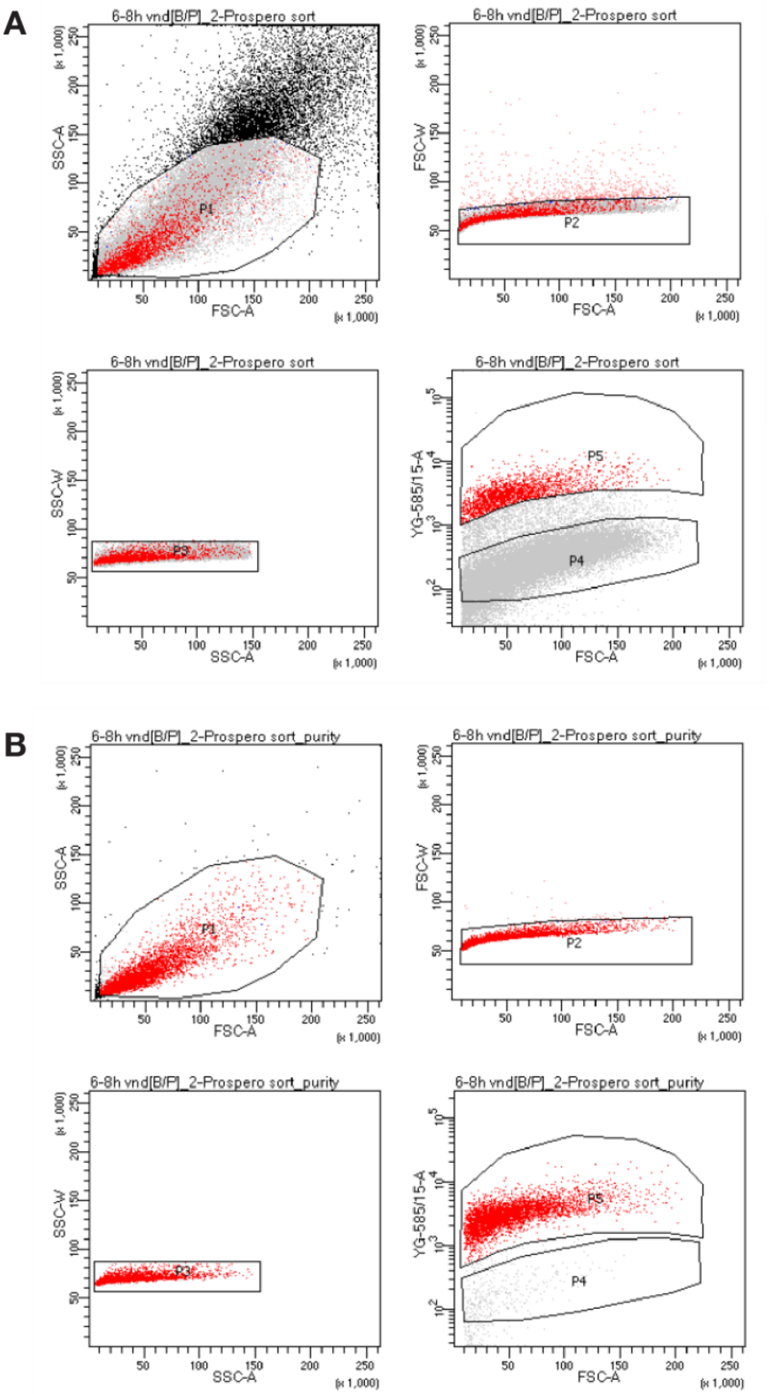

Tube: Prospero sort

Population	#Events	%Parent	%Total
All Events	50,000	####	100.0
P1	34,916	69.8	69.8
P2	34,167	97.9	68.3
P3	34,079	99.7	68.2
P4	26,936	79.0	53.9
P5	2,298	6.7	4.6

Tube: Prospero sort_purity

Population	#Events	%Parent	%Total
All Events	5,556	####	100.0
P1	5,178	93.2	93.2
P2	5,139	99.2	92.5
P3	5,135	99.9	92.4
P4	302	5.9	5.4
P5	4,640	90.4	83.5

Fig. S3: FACS gating strategy yields high purity of sorted populations

(A) Example FACS gating strategy for sorting of marker-positive (P5; 6.7%) and marker-negative cells (P4; 79%). (B) Re-sort of marker-positive sorted cells shows enrichment of marker-positive (P5; 90.4%) and depletion of marker-negative cells (P4; 5.9%).

Development • Supplementary information

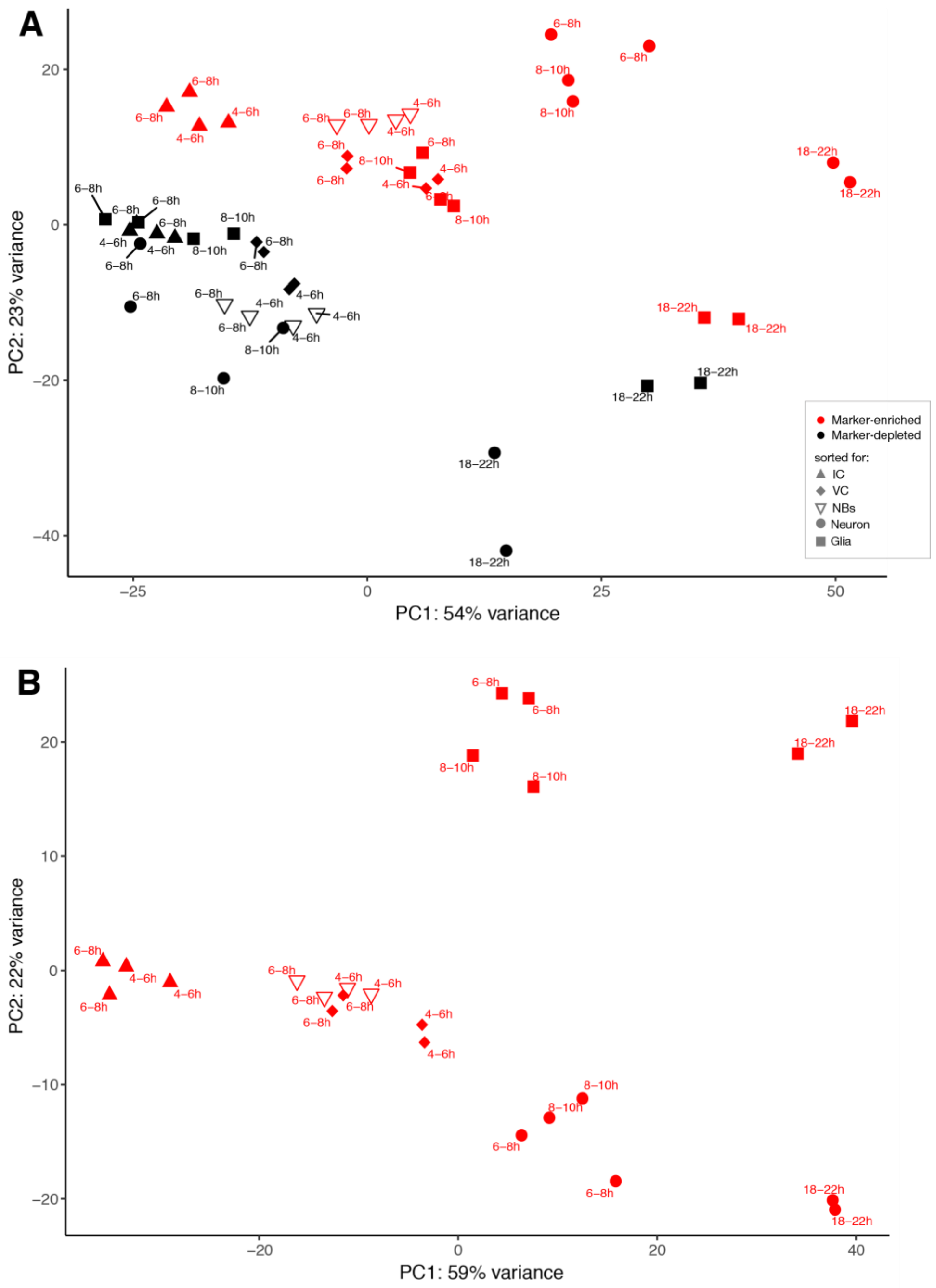
A
PC1: 54% variance
PC2: 23% variance
Marker-enriched
Marker-depleted
sorted for:
IC
VC
NBs
Neuron
Glia
B
PC1: 59% variance
PC2: 22% variance

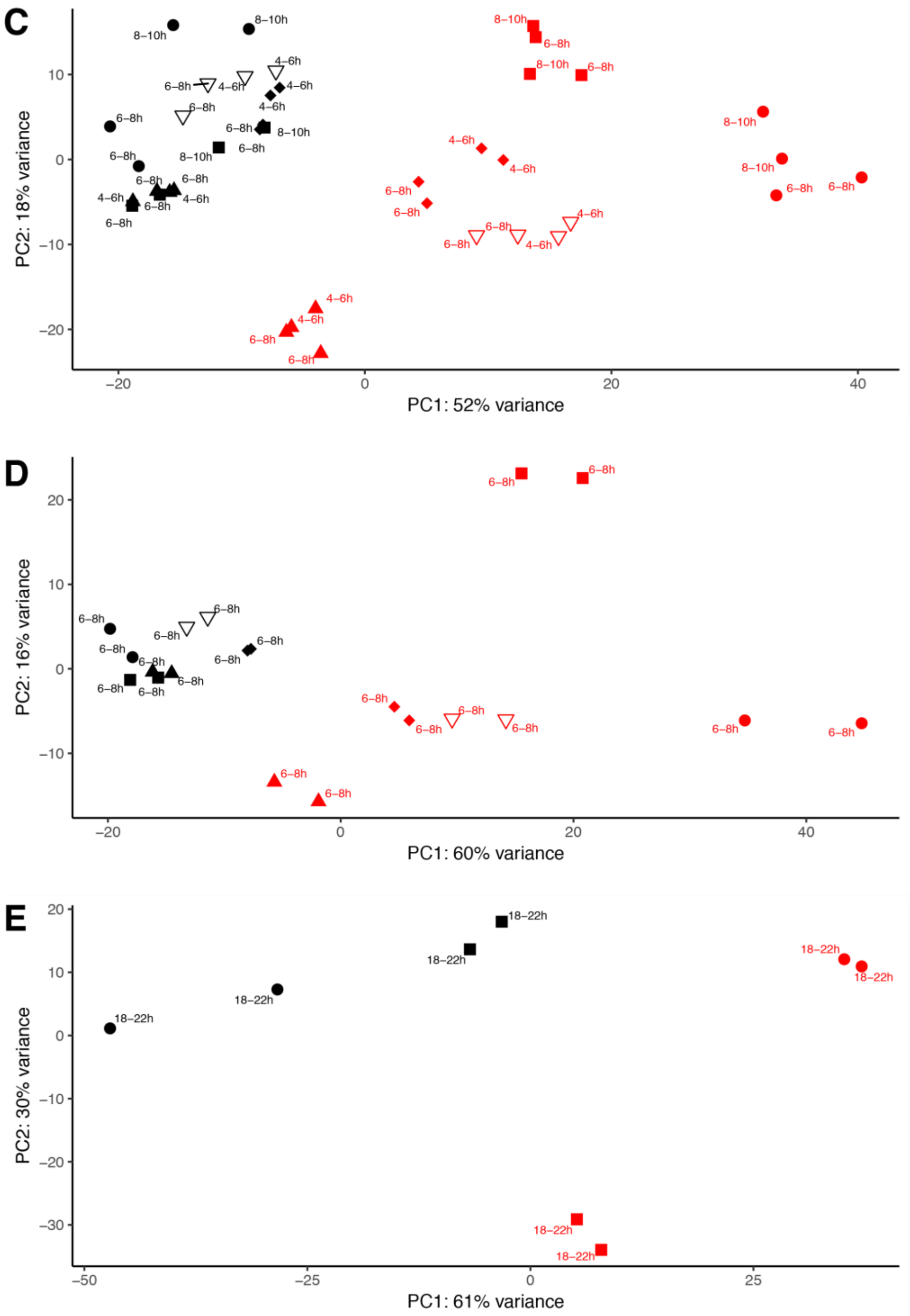
C
PC1: 52% variance
PC2: 18% variance
D
PC1: 60% variance
PC2: 16% variance
E
PC1: 61% variance
PC2: 30% variance

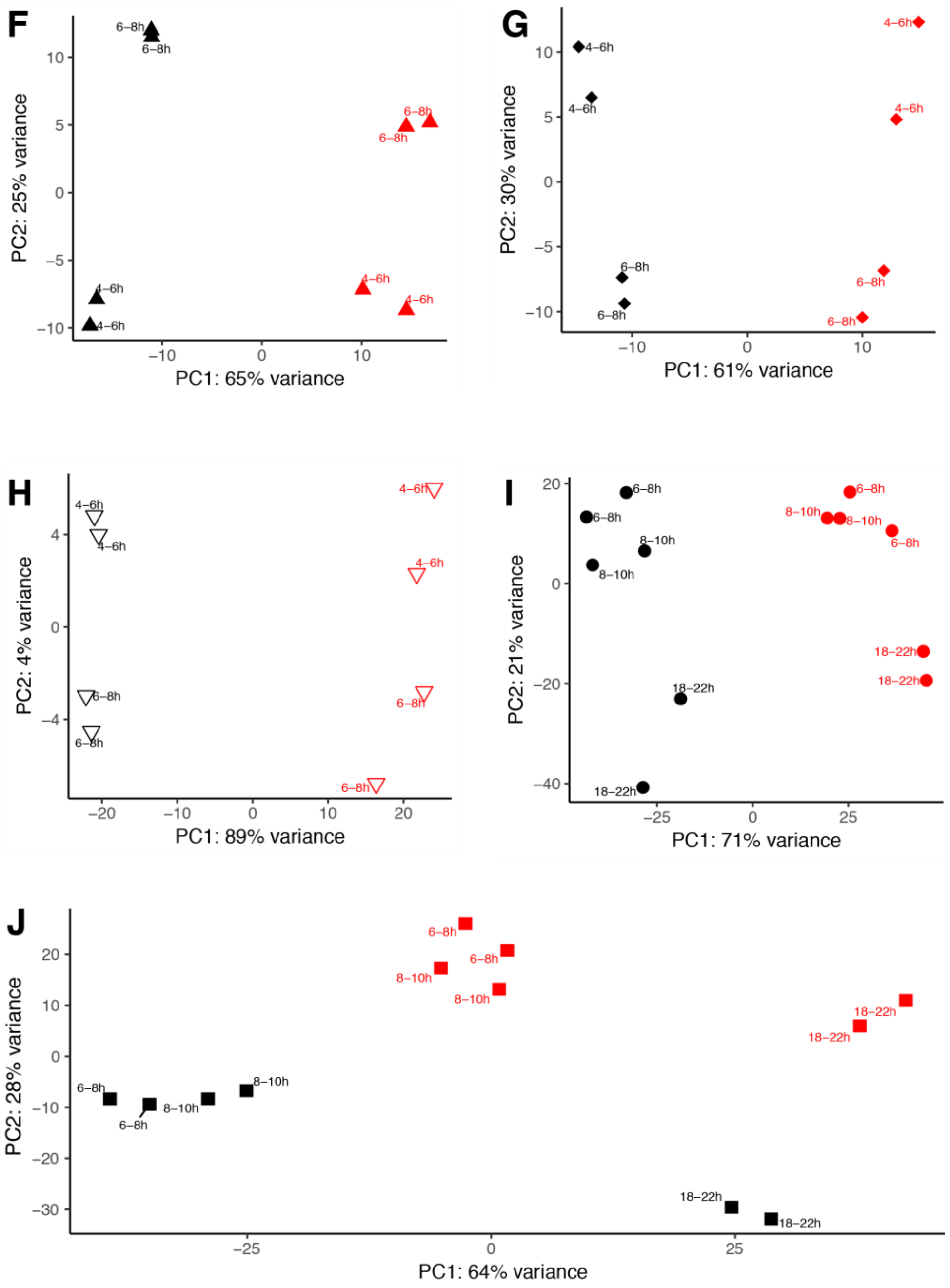

Fig. S4: Principal component analyses separate spatiotemporal transcriptomes according to tissue and developmental age

Principal Component Analysis (PCA) with multiple permutations of datasets generated by *DIV*-SortSeq. Marker-enriched datasets depicted in red, marker-depleted datasets in black. (A) All datasets. (B). Only marker-positive datasets. (C). Only early (4-6, 6-8, 8-10h) datasets. (D) Only 6-8h datasets. (E). Only 18-22h datasets. (F). Only datasets sorted for *ind* expression. (G). All datasets sorted for *vnd* expression. (H) All datasets sorted for *pros* expression. (I) All datasets sorted for *elav* expression. (J) All datasets sorted on *repo* expression.

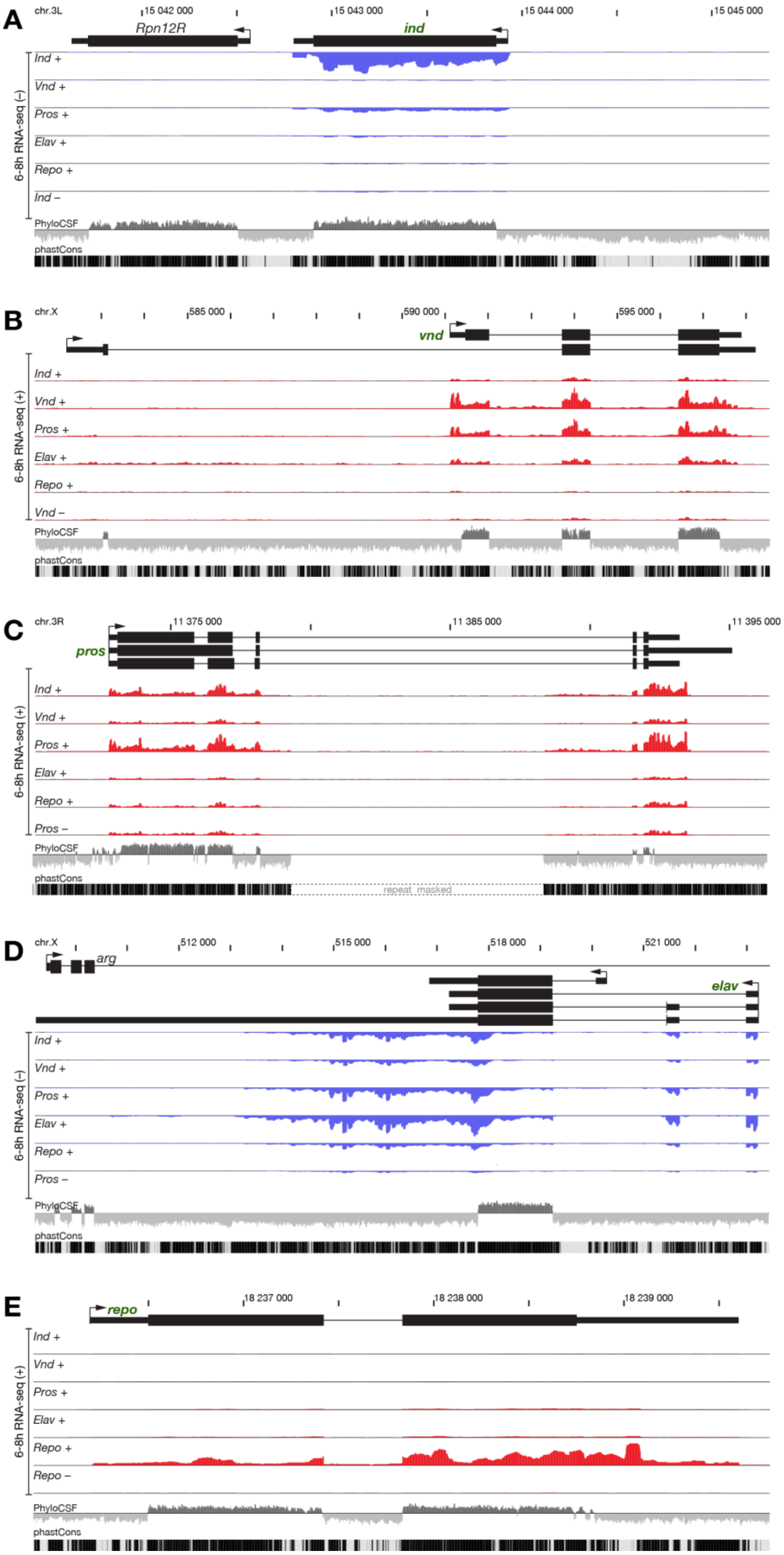

Development • Supplementary information

Fig. S5: Cell type specific transcriptomes at neurogenic marker genes
(A-E) Genome browser data of cell type specific transcriptomes around marker gene loci. Shown are annotated transcripts (top), RNA-seq coverage on the plus- (red) or minus-strand (blue) in the indicated sorts at 6-8hrs AEL, as well as coding potential as measured by PhyloCSF scores (all frames overlaid), and conservation amongst *Drosophilids* (phastCons). (A) *ind* locus. (B) *vnd* locus. (C) *pros* locus. (D) *elav* locus. (E) *repo* locus.

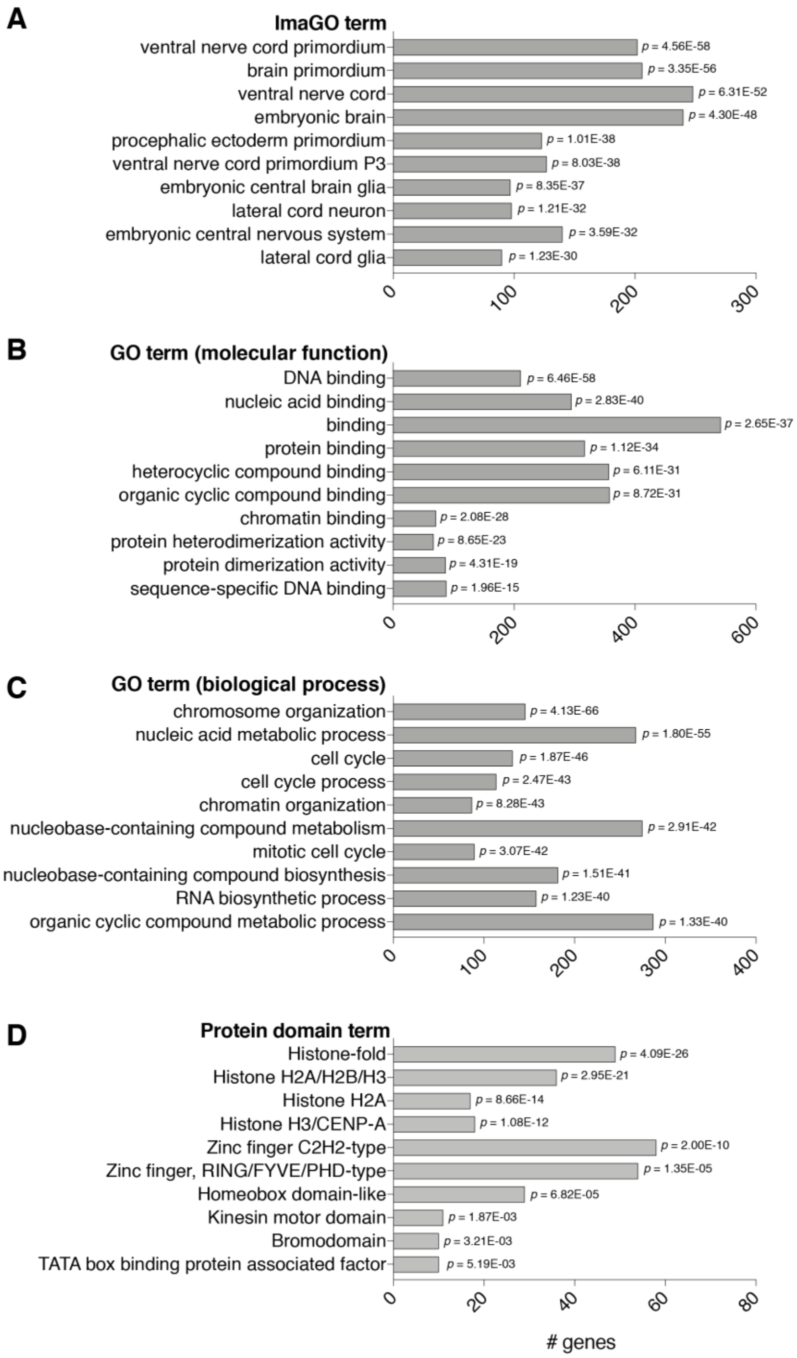

Fig. S6: Annotations of regulated protein-coding genes predict expression and function
Analysis of features of 794 protein-coding genes correlated with known neurogenic genes (Pearson correlation, $r > 0.9$) with Flymine {Lyne:2007jd}. (A) Top ImaGO terms. (B) Top GO terms (molecular function). (C) Top GO terms (biological process). (D) Top protein domain terms. Detailed analysis of all genes can be found in File S2.

Development • Supplementary information

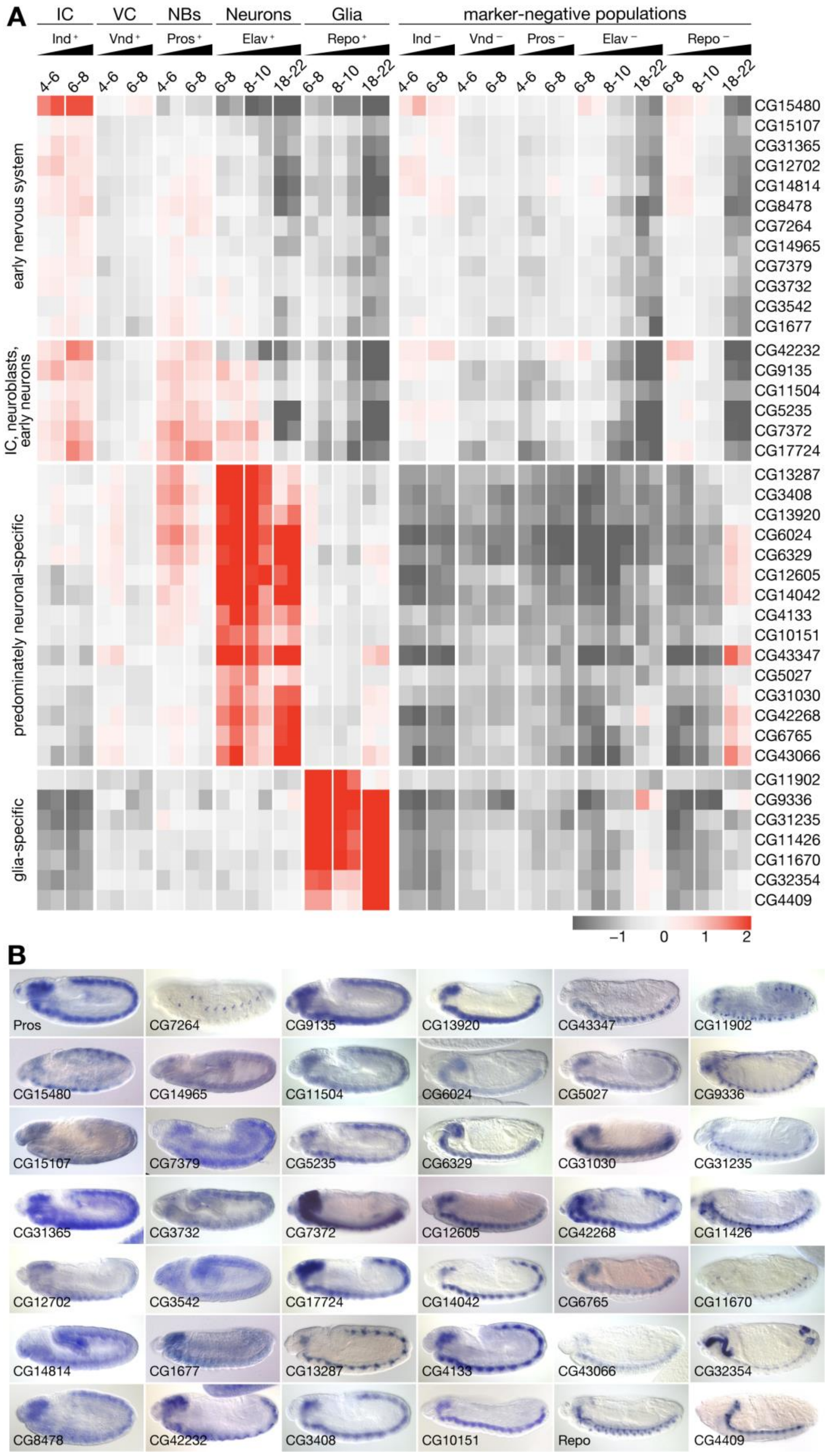

Development • Supplementary information

Fig. S7: DIV-SortSeq reveals a multitude of undescribed genes expressed with neurogenic spatiotemporal specificity

(A) Heatmap of expression profiles of protein-coding genes with unknown function enriched similarly as at least one marker in Fig. 2B (Pearson correlation, $r > 0.9$). Row mean-centered expression values calculated via variance-stabilizing transformation of gene-level RNA-seq counts (scale = $\log_2$ ratio to row mean). (B) Colorimetric RNA *in situ* hybridization of transcripts of unknown function identified in Fig. S7A. All embryos are shown in lateral orientation. Images obtained from Berkeley *Drosophila* Genome Project (Hammonds et al. 2013; Tomancak et al. 2002; Tomancak et al. 2007). A list of all 212 CGs with r>0.9 is available in Table S12.

Development • Supplementary information

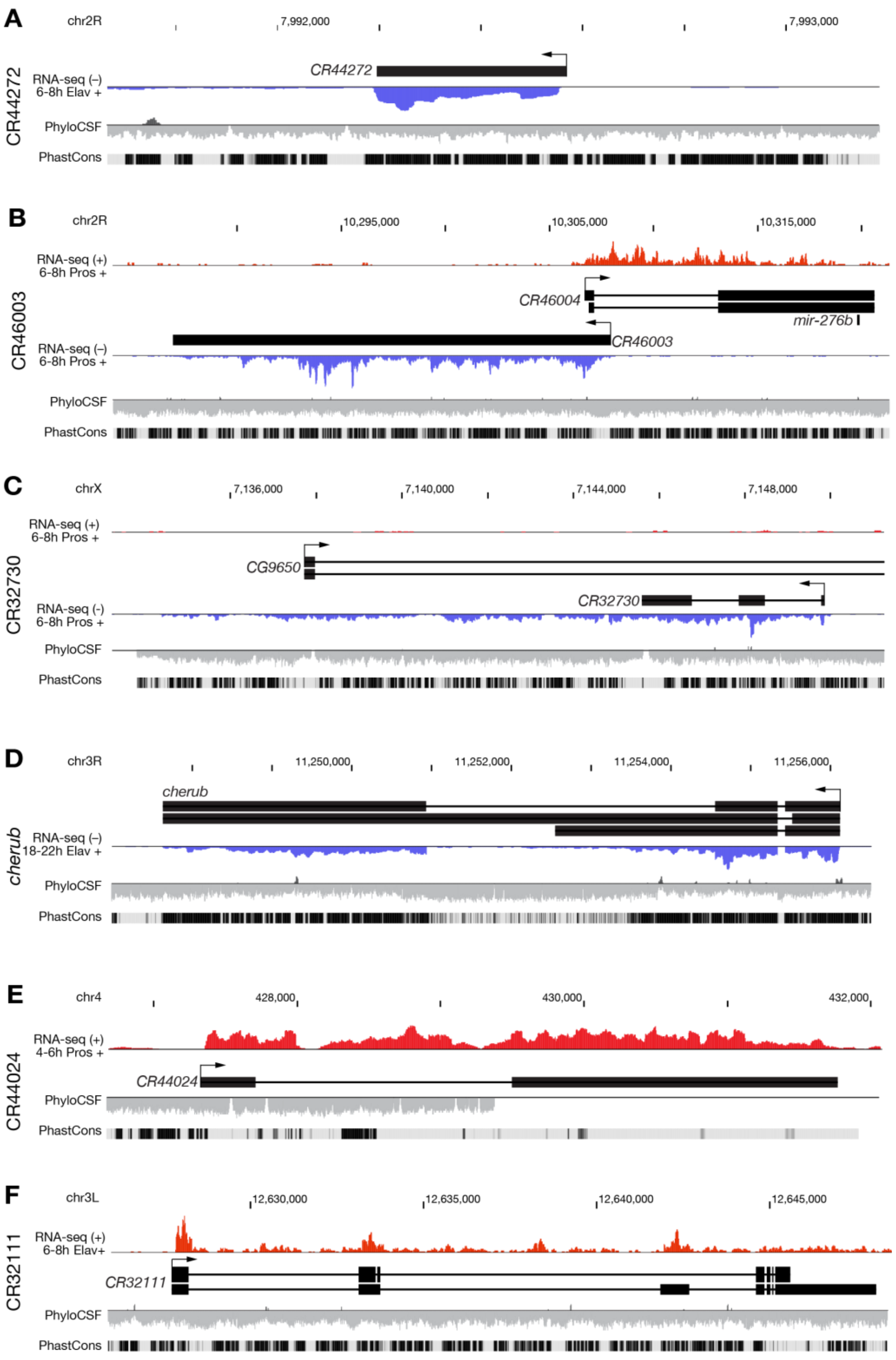

Development • Supplementary information

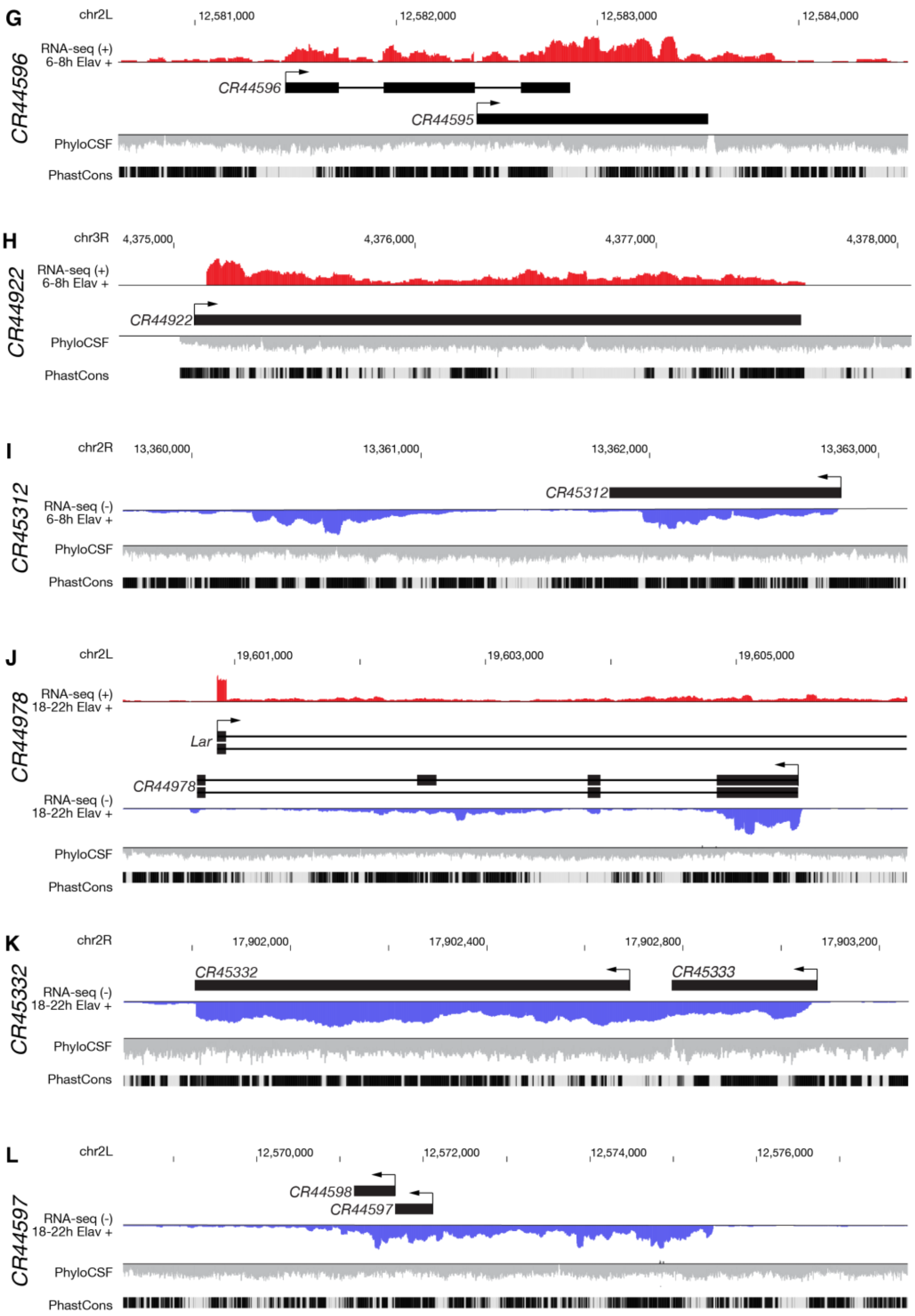

Figure S8: Cell type specific transcriptomes at neurogenic lncRNA loci

Browser images of selected lncRNA loci, display as in Figure S4: The (A) *CR44272,* (B) *CR46003* and *CR46004* (pri-miR-276b), (C) *CR32730,* (D) *cherub,* (E) *CR44024,* (F) *CR32111,* (G) *CR44596,* (H) *CR44922,* (I) *CR45312,* (J) *CR44978,* (K) *CR45332* (This gene model might be misannotated – CR45332 and CR45333 might be a single transcript*),* and the (L) *CR44596* and *CR44597* locus.

Development • Supplementary information

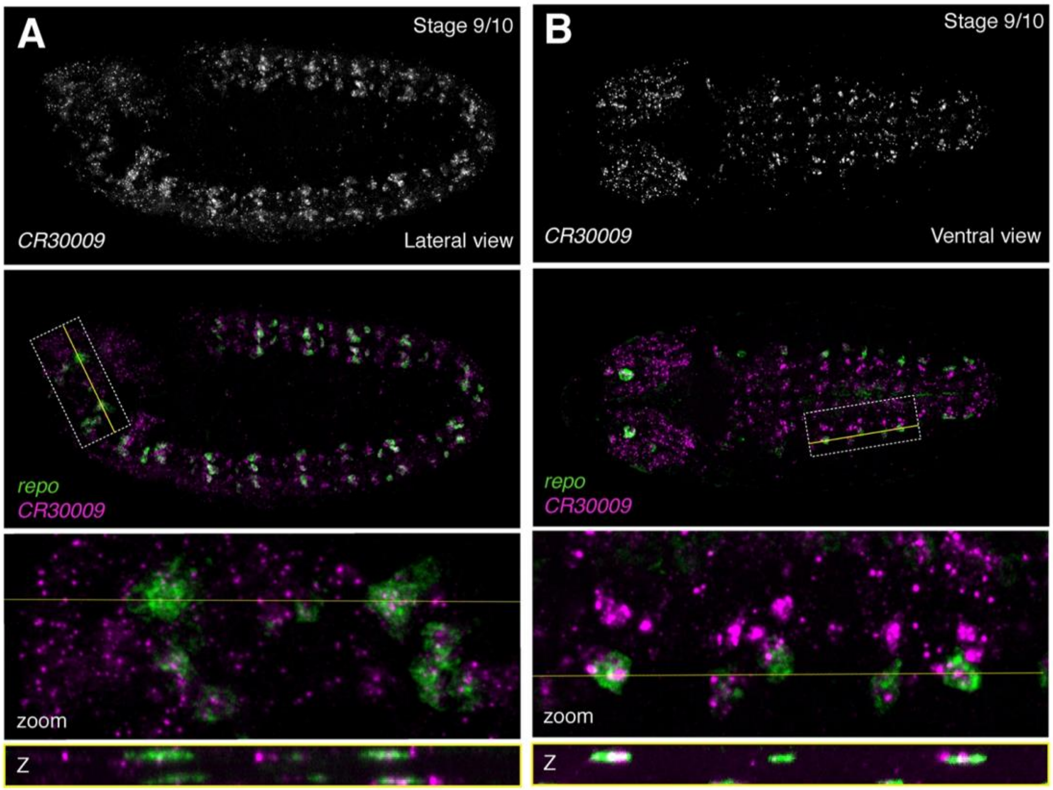

Fig. S9: The lncRNA *CR30009* is expressed in glial subsets in early embryos
RNA fluorescent in situ hybridization (RNA-FISH) against *CR30009* and the glial marker *repo*.
(A) Lateral view, stage 9/10. (B) Ventral view, stage 9/10. Top: *CR30009* alone. Second from top: *CR30009* (magenta) overlaid with *repo* (green). Dashed white box indicates region of interest (ROI) and yellow line indicates Z-slice through ROI. Second from bottom: zoom-in of ROI. Bottom: Slice through Z-stack as indicated by yellow line.

Development • Supplementary information

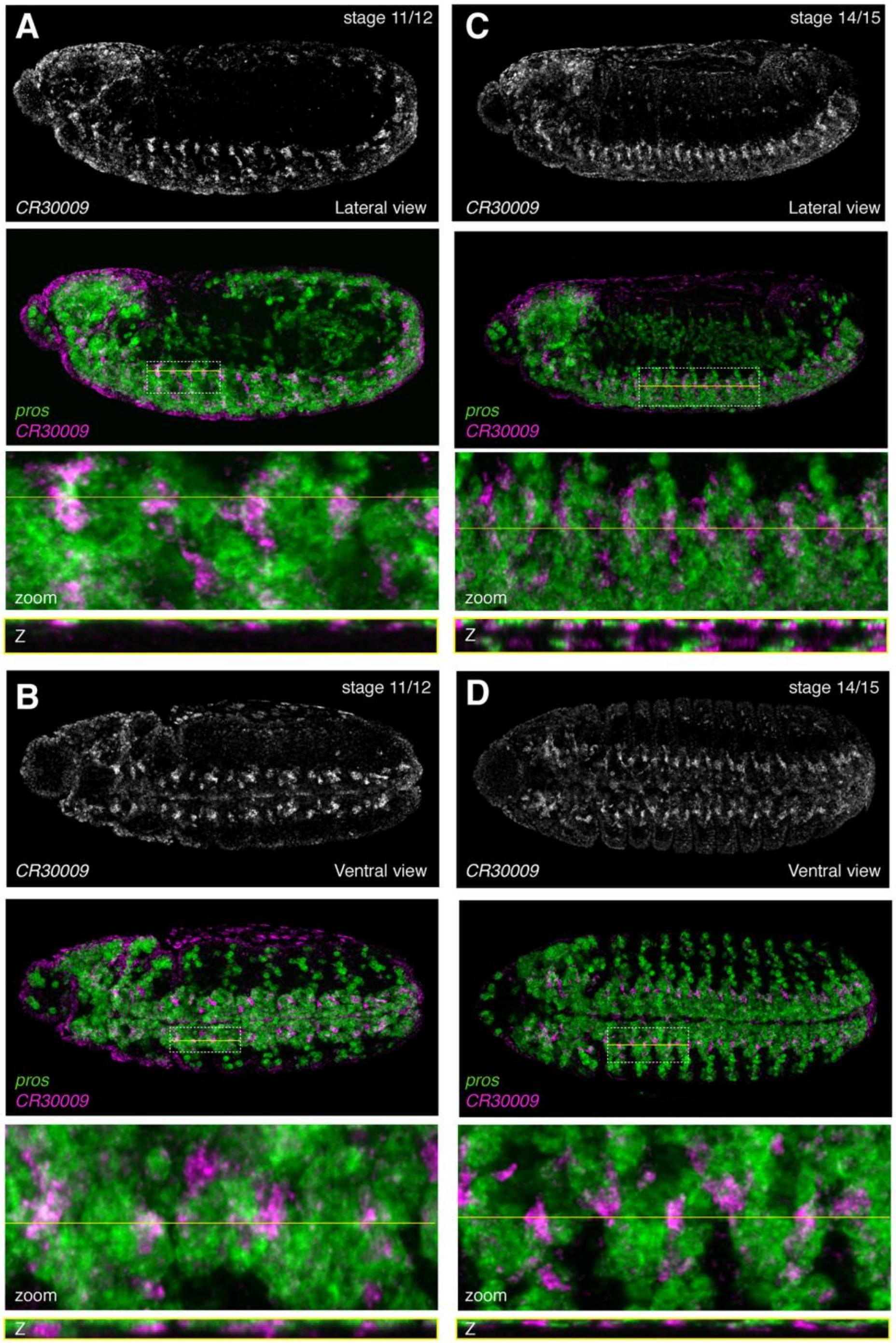

Fig. S10: The lncRNA *CR30009* is expressed in neuroblast subsets

RNA fluorescent in situ hybridization (RNA-FISH) against *CR30009* and the neuroblast marker *pros*. (A) Lateral view, stage 11/12. (B) Lateral view, stage 14/15. (C) Ventral view; stage 11/12. (D). Ventral view; stage 14/15. Top: *CR30009* alone. Second from top: *CR30009* (magenta) overlaid with *pros* (green). Dashed white box indicates region of interest (ROI) and yellow line indicates Z-slice through ROI. Second from bottom: zom-in of ROI. Bottom: Slice through Z-stack as indicated by yellow line.

Development • Supplementary information

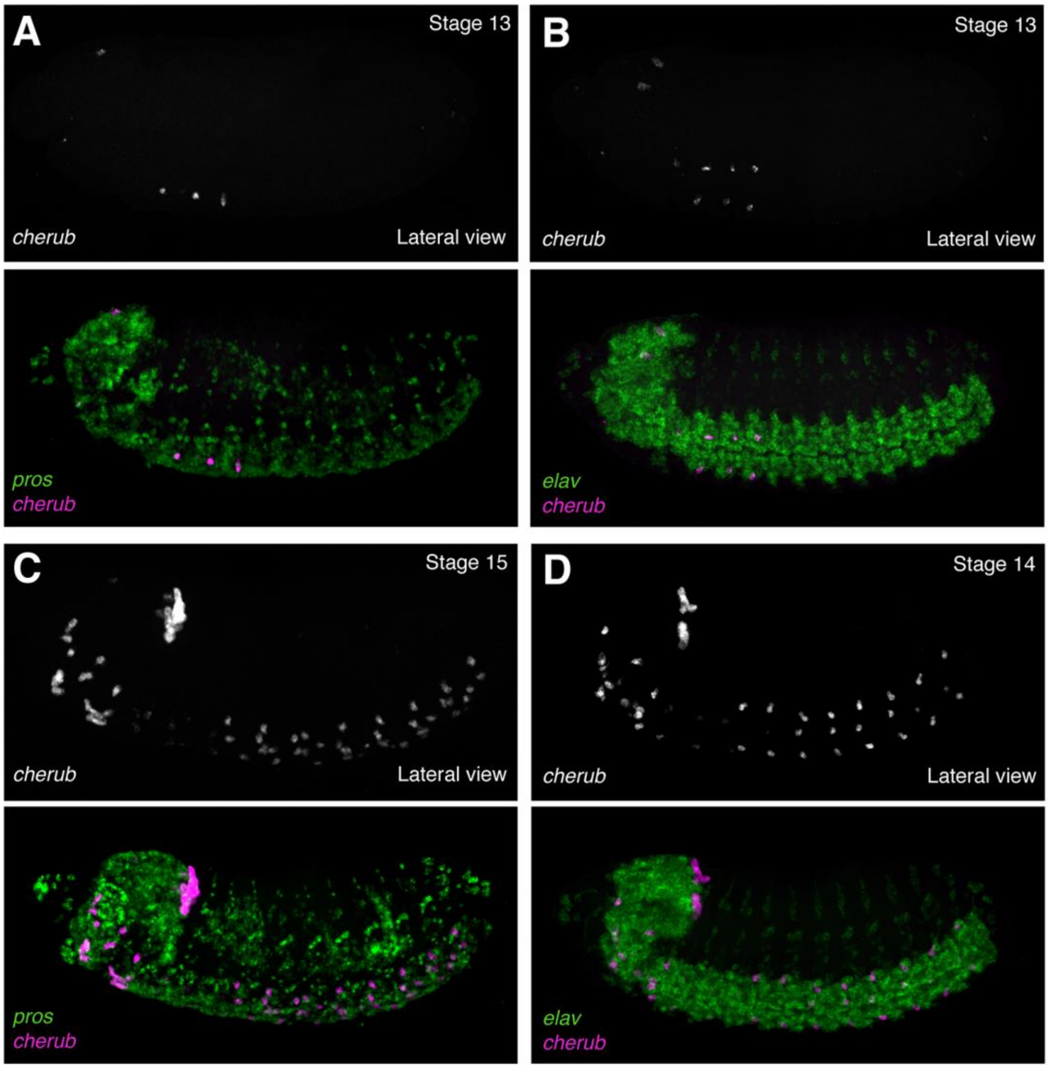

Fig S11: The lncRNA *cherub* is regulated with strict spatiotemporal specificity.
RNA fluorescent in situ hybridization (RNA-FISH) against *cherub*, the neuroblast marker *pros*, and the neuronal marker *elav*. Lateral view. (A) *cherub* with *pros*; stage 13. (B) *cherub* with *elav*; stage 13. (C) *cherub* with *pros*; stage 15. (D) *cherub* with *elav*; stage 14. Top: *cherub* alone. Second from top: *cherub* (magenta) overlaid with marker (green).

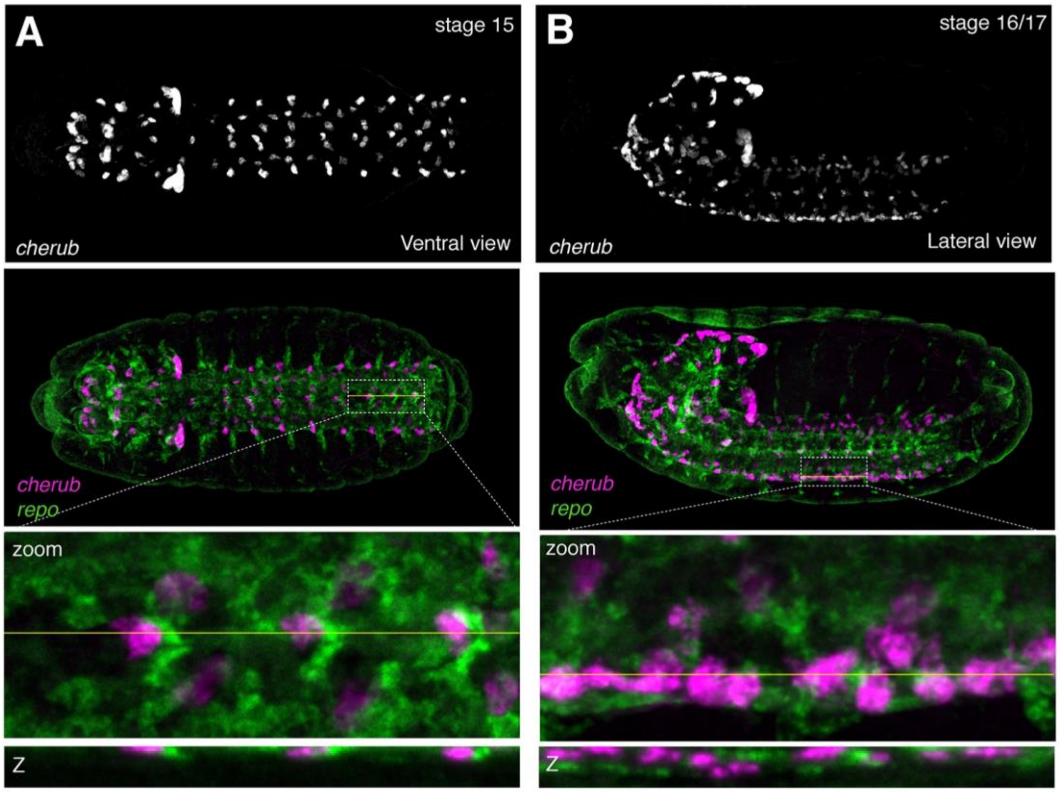

Fig S12: The lncRNA *cherub* is expressed in glial subsets in late embryos

RNA fluorescent in situ hybridization (RNA-FISH) against *cherub* and the glial marker *repo*. (A) Ventral view; stage 15. (B) Lateral view; stage 16/17. Top: *cherub* alone. Second from top: *cherub* (magenta) overlaid with *repo* (green). Dashed white box indicates region of interest (ROI) and yellow line indicates Z-slice through ROI. Second from bottom: zom-in of ROI. Bottom: Slice through Z-stack as indicated by yellow line.

Development • Supplementary information

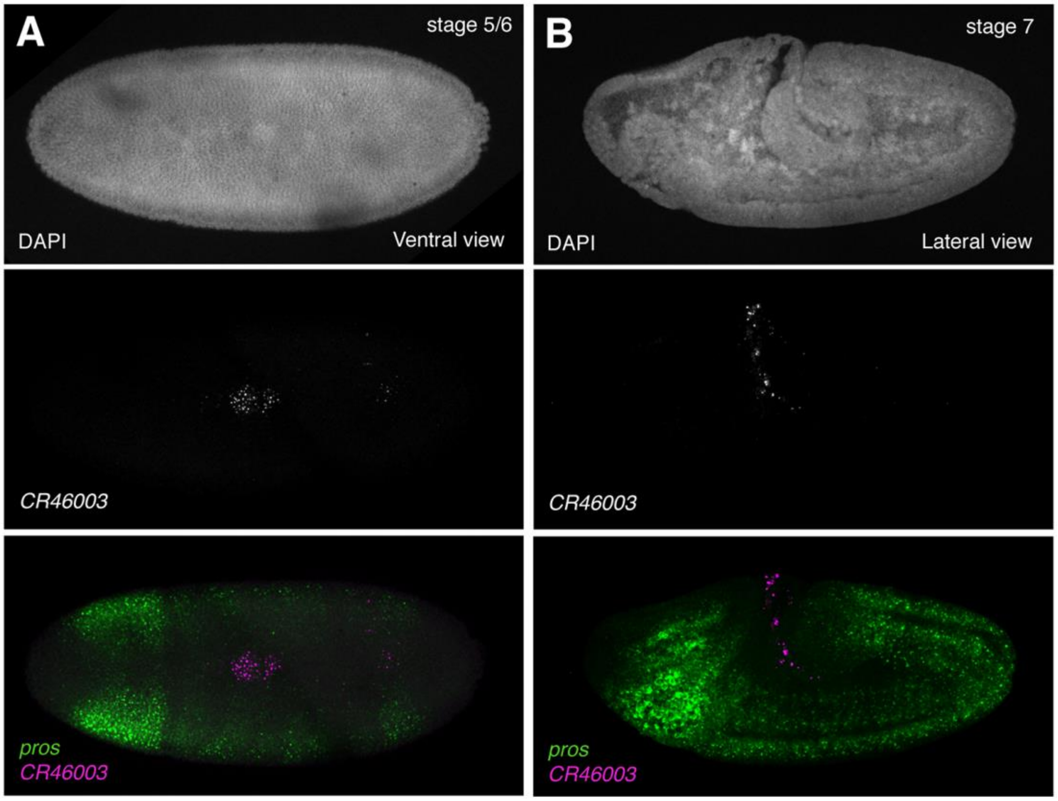

Fig S13: The lncRNA *CR46003* is expressed in early embryogenesis

RNA fluorescent *in situ* hybridization (RNA-FISH) against *CR46003* with the neuroblast marker *pros*. (A) Dorsal view; stage 5/6. (B) Lateral view; stage 7/8. Top: DAPI, middle: *CR46003* alone. Bottom: *CR46003* (magenta) overlaid with *pros* (green).

Development • Supplementary information

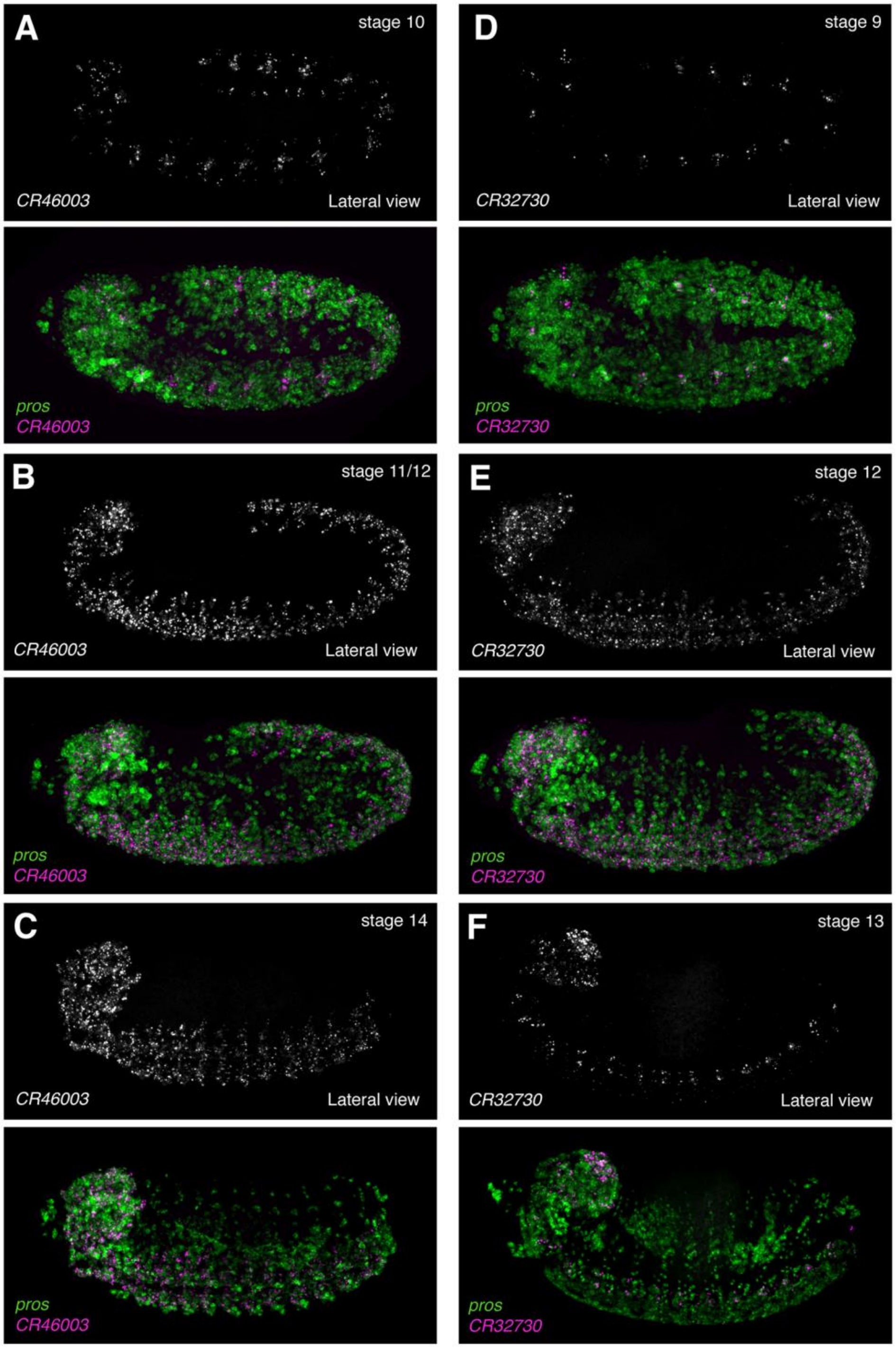

Fig S14: The lncRNAs *CR46003* and *CR32730* are expressed with similar spatiotemporal specificity.
RNA fluorescent *in situ* hybridization (RNA-FISH) against *CR46003* and *CR32730* together with the neuroblast marker *pros*. Lateral view. (A) *CR46003*; stage 10. (B) *CR46003*; stage 11/12. (C) *CR46003*; stage 14. (D) *CR32730*; stage 9. (E) *CR32730*; stage 12. (F) *CR32730*; stage 13. Top: lncRNA alone. Second from top: lncRNA (magenta) overlaid with *pros* (green).

Development • Supplementary information

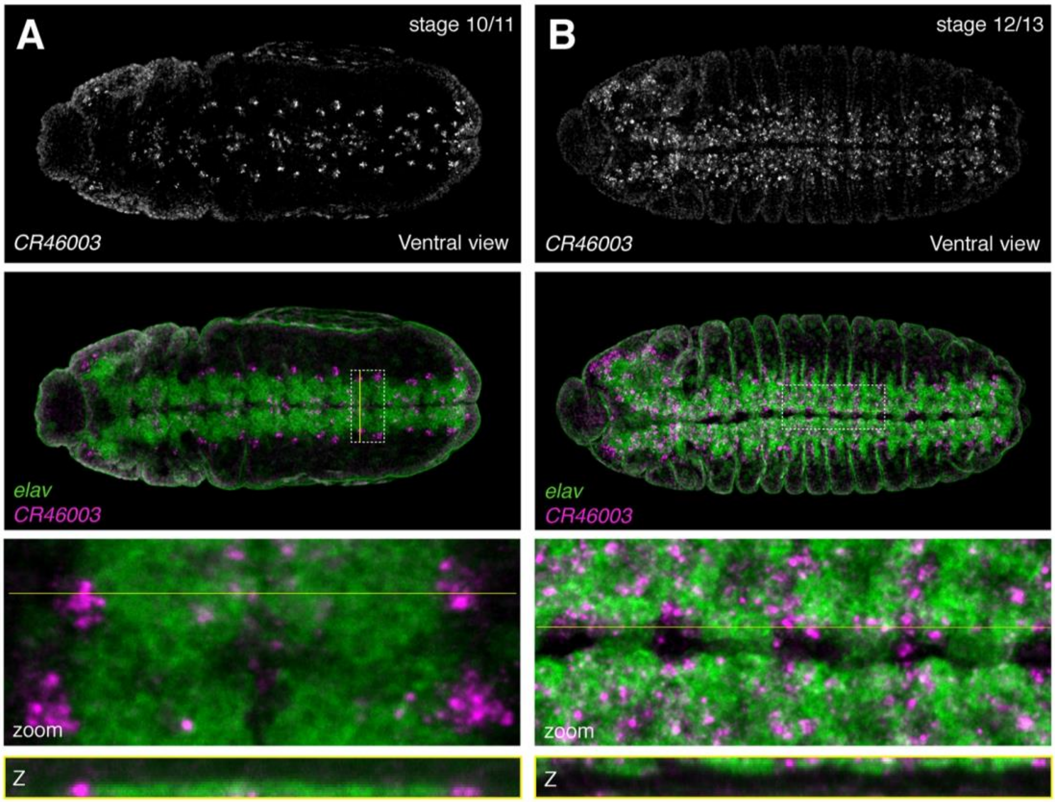

Fig S15: The lncRNA *CR46003* is expressed in neuronal subsets

RNA fluorescent in situ hybridization (RNA-FISH) against *CR46003* with the neuronal marker, *elav*. Ventral view. (A) Stage 10/11. (B) Stage 12/13. Top: *CR46003* alone. Second from top: *CR46003* (magenta) overlaid with *elav* (green). Dashed white box indicates region of interest (ROI) and yellow line indicates Z-slice through ROI. Second from bottom: zom-in of ROI. Bottom: Slice through Z-stack as indicated by yellow line.

Development • Supplementary information

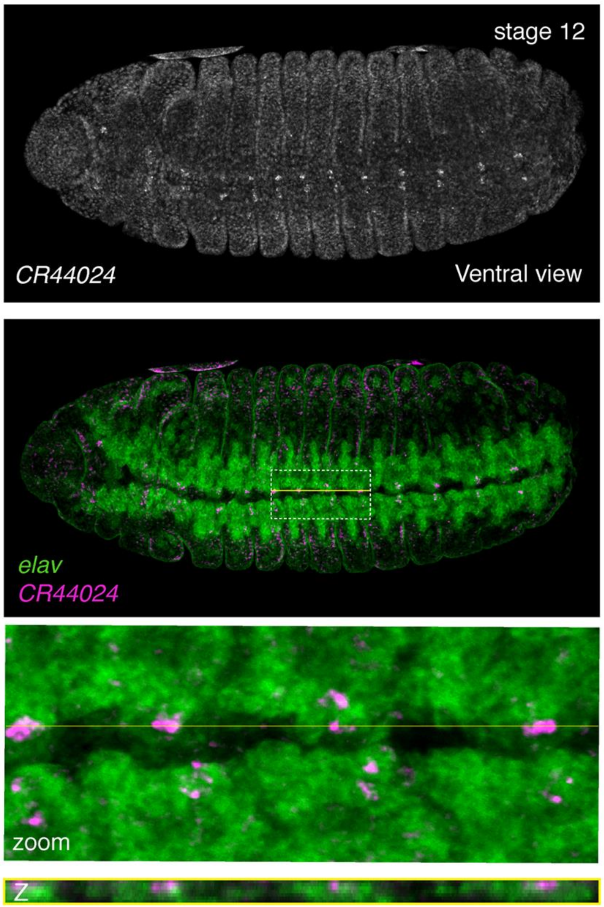

Fig S16: The lncRNA *CR44024* is expressed from stage 12 in neuronal subsets of the ventral nerve cord.

RNA fluorescent in situ hybridization (RNA-FISH) against *CR44024* with the neuronal marker, *elav*. Ventral view, stage 12. Top: *CR44024* alone. Second from top: *CR44024* (magenta) overlaid with *elav* (green). Dashed white box indicates region of interest (ROI) and yellow line indicates Z-slice through ROI. Second from bottom: zom-in of ROI. Bottom: Slice through Z-stack as indicated by yellow line.

Development • Supplementary information

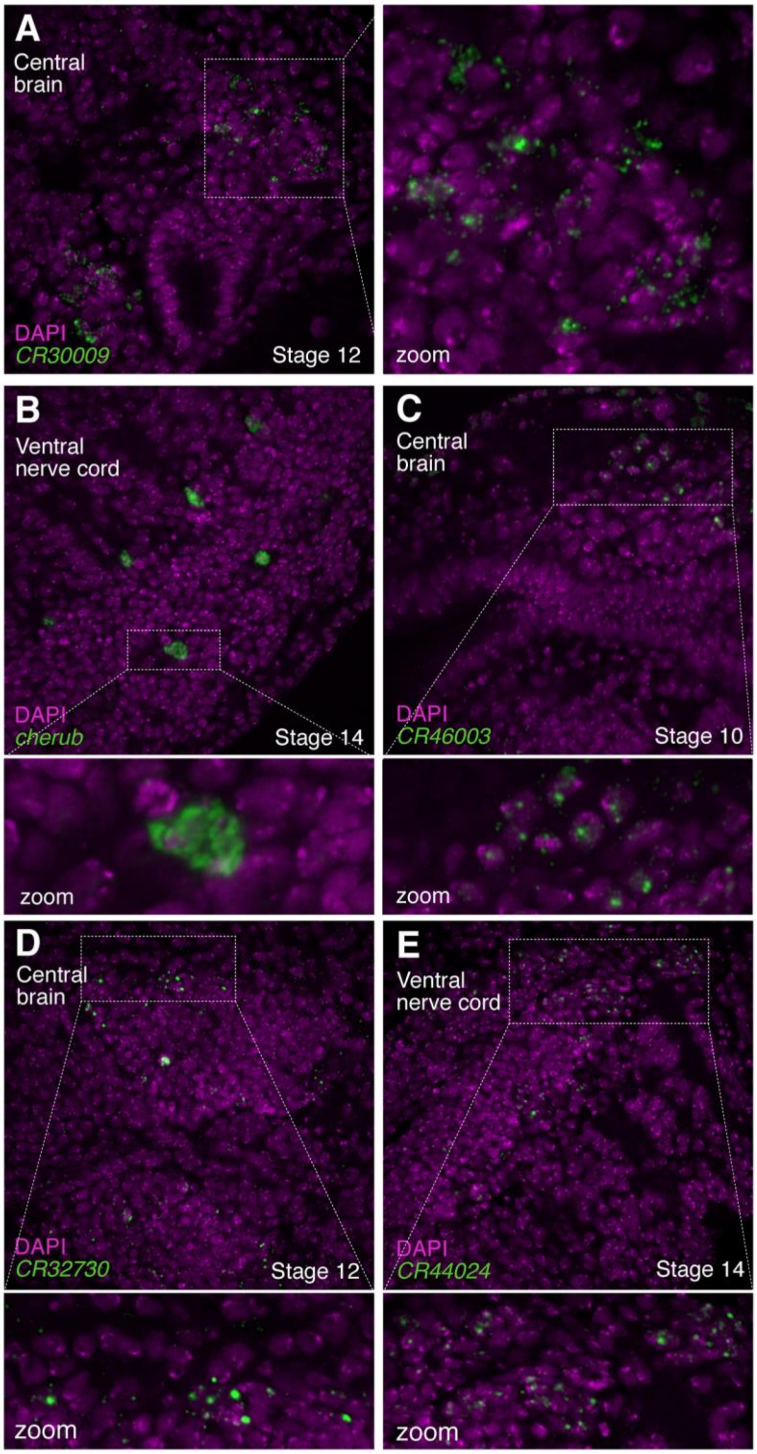

Fig S17: LncRNAs exhibit varying patterns of subcellular distribution

RNA fluorescent in situ hybridization (RNA-FISH) against lncRNAs (green) overlaid with the nucleic acid marker, DAPI (magenta). (A) *CR30009*; central brain, stage 12. (B) *cherub*; ventral nerve cord, stage 14. (C) *CR46003*; central brain, stage 10. (D) *CR32730*; central brain, stage 12. (E) *CR44024;* ventral nerve cord, stage 14. (A) Left panel: Dashed white box indicates region of interest (ROI). Right panel: zom-in of ROI. (B-E) Top panel: Dashed white box indicates region of interest (ROI). Bottom panel: zoom-in of ROI.

Development • Supplementary information

Supplementary References

Barolo, S., Castro, B. & Posakony, J.W., 2004. New Drosophila transgenic reporters: insulated P-element vectors expressing fast-maturing RFP. *BioTechniques*, 36(3), pp.436–40– 442.

Bhatt, D.M. et al., 2012. Transcript dynamics of proinflammatory genes revealed by sequence analysis of subcellular RNA fractions. *Cell*, 150(2), pp.279–290.

Hrvatin, S. et al., 2014. MARIS: Method for Analyzing RNA following Intracellular Sorting K. Aalto-Setala, ed. *PLoS ONE*, 9(3), pp.e89459–6.

Karaiskos, N. et al., 2017. The Drosophila embryo at single-cell transcriptome resolution. *Science*, 358(6360), pp.194–199.

Markstein, M. et al., 2004. A regulatory code for neurogenic gene expression in the Drosophila embryo. *Development*, 131(10), pp.2387–2394.

Pawlicki, J.M. & Steitz, J.A., 2008. Primary microRNA transcript retention at sites of transcription leads to enhanced microRNA production. *The Journal of cell biology*, 182(1), pp.61–76.

Rosner, M. & Hengstschläger, M., 2012. Detection of cytoplasmic and nuclear functions of mTOR by fractionation. *Methods in molecular biology (Clifton, N.J.)*, 821(Chapter 8), pp.105–124.

Rubin, G.M. & Spradling, A.C., 1982. Genetic transformation of Drosophila with transposable element vectors. *Science*, 218(4570), pp.348–353.

Schmittgen, T.D. & Livak, K.J., 2008. Analyzing real-time PCR data by the comparative C(T) method. *Nature Protocols*, 3(6), pp.1101–1108.

Shechner, D.M. et al., 2015. Multiplexable, locus-specific targeting of long RNAs with CRISPR-Display. *Nature Methods*, 12(7), pp.664–670.

Stapleton, M. et al., 2002. The Drosophila gene collection: identification of putative full-length cDNAs for 70% of D. melanogaster genes. *Genome Research*, 12(8), pp.1294–1300.

Vandesompele, J. et al., 2002. Accurate normalization of real-time quantitative RT-PCR data by geometric averaging of multiple internal control genes. *Genome biology*, 3(7), p.RESEARCH0034.

Development • Supplementary information
